# Exploring a Dynamic Template Matching Algorithm for the Automatic Extraction of P3 Latencies

**DOI:** 10.1111/psyp.70212

**Published:** 2025-12-23

**Authors:** Sven Lesche, Kathrin Sadus, Anna‐Lena Schubert, Christoph Löffler, Dirk Hagemann

**Affiliations:** ^1^ Institute of Psychology Heidelberg University Heidelberg Germany; ^2^ Institute of Psychology University of Mainz Mainz Germany

**Keywords:** event‐related potentials, latency extraction, P3, template matching

## Abstract

In this study, we explore a novel template matching algorithm using the grand average as a dynamic template to extract P3 latencies. This new algorithm outperforms peak latency and fractional area latency algorithms in both empirical as well as simulated data. A modified fractional area latency algorithm proposed by Liesefeld (2016, 2018) performed best among all previously employed approaches. It matched the performance of the template matching algorithms in the empirical data, but performed worse in the simulation. Template matching algorithms showed high agreement (ICC = 0.89) with latencies extracted by expert researchers and the most accurate recovery of simulated latency shifts (ICC = 0.91). Our results highlight the robustness of template matching algorithms across various tasks, preprocessing steps, and algorithm hyperparameters. Additionally, template matching provides a fit statistic that researchers can use to automatically discard ERPs with poor matches or flag certain ERPs for manual review. This fit statistic allows targeted manual intervention, increasing the efficiency and objectivity of latency extraction. Overall, the straightforward application of our template matching algorithm allows it to be easily integrated into multiverse studies or automated pipelines.

## Impact Statement

The template matching algorithm introduced in this paper offers a robust, objective, and efficient method for extracting individual differences in P3 latencies, outperforming traditional approaches like peak and fractional area latency methods. By providing accurate latency recovery, high validity across varied conditions, and a fit statistic for quality control, it enhances reliability and automation in ERP analysis. Its straightforward implementation makes template matching ideal for multiverse studies and automated pipelines, advancing reproducibility and efficiency in ERP research.

## Introduction

The latency of event‐related potentials (ERPs) is a valuable measure of the speed of neurocognitive operations. This measure has been used to investigate the effect of experimental manipulations on neurocognitive processes or to study individual differences in mental speed and has provided substantial insight into brain processes underlying cognitive functions. For example, a latent factor comprising individual latencies of the N2, P2, and P3 components in simple decision tasks has been shown to explain 80% of the variance in general intelligence (Schubert et al. 2017). However, testing differences between conditions or measuring individual differences in the latency of components requires the extraction of latency estimates on the individual subject level. Such research based on individual estimates of latency requires extraction methods that provide consistently reliable and valid measures to ensure the replicability of results. So far, manual extraction of P3 latencies has yielded the most reliable and valid measures (Sadus et al. 2024; Schubert et al. 2023). However, manual extraction impairs objectivity and is inefficient, especially in the face of large data. Algorithms aiming to automate latency extraction, such as peak latency algorithms or fractional area latency algorithms, are time‐effective and objective, but often lack in reliability or validity (Kiesel et al. 2008; Sadus et al. 2024; Schubert et al. 2023). This paper introduces a novel algorithm for the automatic extraction of P3 latencies based on template matching. We aim to show that this new algorithm improves on existing approaches such as peak latency or fractional area latency and enables more efficient, objective, reliable, and valid extraction of ERP latencies.

In this paper, we chose to restrict our analysis to the P3 and provide proof‐of‐concept for this component before extending our work to other ERP components. Specifically, we will be limiting our analysis to the P3b (Verleger 2020). Future mention of “P3” always refers to the parietal P3b. The P3 is one of the most widely used ERP components and is characterized by a positive‐going deflection around 300–500 ms after stimulus onset (Donchin 1981; Luck 2014). It is related to higher‐order cognitive processing and is often associated with updating of information (Polich 2007), stimulus classification (Duncan‐Johnson 1981), response selection (Polich 2007), and stimulus–response link reactivation (Verleger 2020). P3 latencies have been shown to be helpful for explaining individual differences in intelligence (Hilger et al. 2022; Schubert et al. 2017, 2023). Given the widespread use of the P3 and its connection to numerous significant findings, we decided to concentrate on this ERP component in the present paper.

### 1 Latency Extraction Algorithms

The two approaches most commonly used to automatically extract component latencies are either based on the maximum voltage deflection within a fixed measurement window (peak latency) or the area under the ERP signal (fractional area latency; Luck 2014). Peak latency algorithms are based on the assumption that the point in time where the maximum voltage deflection occurs is the best estimate of the true latency of the underlying component. However, high voltage deflections are sensitive to high frequency noise and the superimposed signal of surrounding components (Luck 2014). This influence of unrelated sources on the maximum voltage deflection challenges the central assumptions of peak latency algorithms (Luck 2014). Additionally, Kiesel et al. (2008) presented an empirical challenge to peak latency approaches. They showed that the peak latency algorithm was unable to accurately recover simulated latency differences across a range of different components, including the P3. In a recent multiverse study by Schubert et al. (2023), the authors were not able to apply automated latency extraction tools as they lead to unreliable latency estimates. In this study and previous studies of our lab, manual oversight and latency extraction provided reliable estimates and high correlations with intelligence (Schubert et al. 2023, 2017) indicating that manual extraction yielded valid estimates of ERP latency. Sadus et al. (2024) conducted a multiverse study to test the psychometric properties of varying extraction strategies more formally and demonstrated considerable issues in the extraction of P3 latencies using automated approaches. In their multiverse study with varying pre‐processing steps and experimental tasks, the peak latency algorithm often produced low reliabilities, showed poor homogeneity, and resulted in questionable effect size estimates. Only manual extraction of individual P3 latencies proved to yield measures that were consistently reliable and valid. Taken together, these methodological issues and empirical findings demonstrate that the peak latency algorithm is not a sufficiently reliable and valid algorithm that could replace manual quantification of ERP latencies.

Fractional area latency algorithms hope to remedy some of the methodological issues associated with the peak latency approach. These algorithms estimate the latency of a component by calculating the area between the signal and a horizontal axis—typically the time axis—within a defined measurement window. Component latency is specified as the time that divides this area into a given fraction. The 50% area latency algorithm, for example, finds the time point that divides the area—the integral under the curve between two time points—into two halves. Fractional area latency algorithms are unaffected by high‐frequency noise (Luck 2014). Nonetheless, they remain highly sensitive to the measurement window specified by the researcher (Luck 2014). A wider measurement window may include signal from surrounding components, while a shorter measurement window may capture only a part of the signal of the component of interest. Compared to peak latency algorithms, fractional area latency algorithms showed better recovery of simulated latency differences but also failed to consistently recover simulated experimental effects (Kiesel et al. 2008). Similarly, Sadus et al. (2024) found that fractional area latency algorithms were unable to consistently produce good reliabilities, homogeneities, and plausible effect sizes when extracting P3 latencies.

It should be noted that according to Kiesel et al. (2008), the combination of jackknifing and the fractional area latency algorithm leads to the best recovery of simulated experimental effects in P3 latencies. In contrast, Sadus et al. (2024) found that jackknifing may lead to implausible effect sizes, especially in large data sets. Therefore, we will not include jackknifing in our analysis. With large numbers of participants, jackknifed sub‐averages are highly similar (or nearly identical) and thus artificially decrease the variance of the extracted latencies. Even after correcting for this deflation of variance and applying a transformation to recover individual subject latencies (Smulders 2010), jackknifing failed to consistently produce acceptable reliabilities and effect sizes (Sadus et al. 2024).

Due to the issues of peak latency algorithms, fractional area latency algorithms, and jackknifing procedures, manually extracting component latencies seems to remain the best approach regarding reliability and validity when individual differences are of interest (Sadus et al. 2024). In this study by Sadus et al. (2024), all of the measurement pipelines that consistently produced desirable psychometric properties included manual extraction of component latencies. However, manual extraction of latencies suffers from poor objectivity and low efficiency. An extraction algorithm that is sufficiently reliable and valid would greatly improve objectivity and efficiency.

### 2 Template Matching Approach

We aim to present an algorithm that can match the reliability and validity of human performance, while improving objectivity and efficiency. With the intention of matching human performance, we sought to embed some of the strategies used by human researchers into the latency extraction algorithm. This section will outline the general template matching approach. Detailed information is provided in the methods section.

Human researchers may be better at extracting ERP latencies because they use more information than traditional extraction algorithms. Before even investigating individual subject ERPs, most researchers inspect the grand average in order to better understand the prototypical ERP structure in their specific task, condition, and subject sample. When extracting the latency of subject‐level ERPs, they are then looking to identify the part of the signal that most closely resembles the component of interest in the grand average. This might not always be an exact match, because amplitudes for the individual might be higher or the component might appear later compared to the grand average, but the general shape of the component of interest will still be present. Expert researchers identify the part of the individual signal that best resembles the grand average component and then extract the subject's latency accordingly.

Similarly, our algorithm finds the closest match of the grand average to the individual subject‐level ERPs. To achieve this, we based our new algorithm on the concept of template matching. We use the grand average over all subjects as a template, which includes an idealized version of the component of interest. Then, the algorithm uses this template to identify the P3 in subject‐level ERPs by figuring out which part of the ERP best reflects the structure in the template. This mirrors the process expert ERP researchers use to extract individual latencies and allows the algorithm to use more of the available information.

**FIGURE 1:**
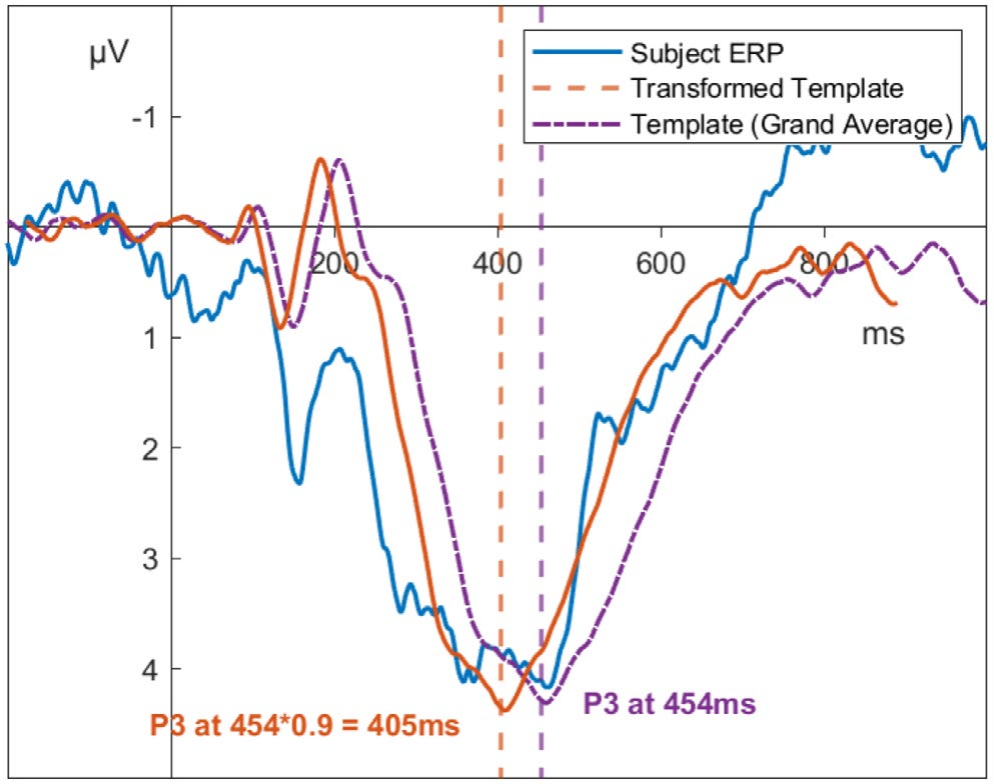
Transforming the template to match an ERP. Illustration of a transformation of the grand average as a template. The grand average (purple) peaks at 454 ms. To maximize the similarity to the subject level ERP, the template is compressed along the time‐axis by a factor of 0.9. This yields the measurement of the subject‐level latency at 405 ms.

Using the grand average as a template is ideal as it contains the average latency and average amplitude of a particular component. Nonetheless, subject‐level ERPs differ from this grand average in both latency and amplitude. Quantifying these individual differences is the goal of our algorithm. With an estimate of the latency of the component of interest in the grand average and an estimate of the difference between the subject‐level ERP and the grand average, the algorithm can recover individual latencies. To quantify individual differences in relation to the grand average, we use a dynamic template with variable latency and amplitude instead of only using the static grand average. This dynamic template is derived from the grand average by adding two free parameters that allow compressing or stretching both the latency and amplitude of the original grand average. Our algorithm then determines which transformed template shows the best fit to the subject‐level ERP. For example, if the template amplitude needs to be stretched by a factor of 1.1 and its latency multiplied by 0.9 to best fit the subject‐level ERP, then the subject‐level latency is quantified as 0.9 times the grand average's component latency (see Figure 1).

Optimization of these free parameters is based on so‐called *similarity measures*. The similarity measure quantifies the fit of the transformed template to the subject‐level signal. Different approaches to similarity measures are present in the template matching literature and fall into one of two camps: distance‐based or correlation‐based (Brunelli 2009; Mahalakshmi et al. 2012). These different approaches may result in different performances of the template matching algorithm (Brunelli and Poggiot 1997; Goshtasby et al. 1984). We therefore implemented a version of both types of similarity measures. The first is based on minimizing the squared distance (MINSQ) and the second is based on maximizing the correlation between the transformed template and the signal (MAXCOR).

If used without any further modification, all points of the signal would contribute equally to the similarity measure. However, as the component of interest in the grand average only consists of a part of the signal and does not incorporate all points in time, we tested the use of weighting windows that weigh the similarity measure depending on the time of the component of interest in the grand average. When extracting P3 latencies, which usually occur around 300‐500 ms (Luck 2014), earlier and later activity in the grand average should not influence the template matching procedure as much as the signal central to the P3. Using these weighting windows helps the algorithm to more closely mirror human behavior. Similarly, researchers manually extracting latencies focus on those parts of the signal where the component of interest occurs.

Both peak and area latency algorithms also focus on specific segments of the signal, determined by the use of measurement windows. Activity outside of these fixed windows is not considered in these algorithms. These measurement windows are applied to each subject‐level ERP, directly influencing which part of the signal is considered. In contrast, our template matching algorithm uses windows based on the grand average, which do not directly define the part of the subject‐level signal being considered. Instead, they influence the template that is used during the template matching algorithm. To underline this difference, we will refer to windows set in the context of the template matching algorithm as *weighting windows*.

These weighting windows form the basis of a weighting vector used during the template matching procedure. Generally speaking, the difference (or correlation) between template and signal outside of that window is less important than the difference inside of the window.

### 3 The Present Study

We used the EEG‐data from the study by Löffler et al. (2024), which were also used by Sadus et al. (2024), and investigated both the psychometric properties of our new algorithm as well as the correlation with manually extracted latencies. This allows us to evaluate whether our new algorithm is a suitable replacement for manual extraction. Additionally, using data from this multiverse study allows us to investigate the impact of different tasks and pre‐processing steps on the evaluated algorithms and provide insight into the conditions under which each algorithm performs best or worst.

We expanded this validation approach by a simulation study based on data used by Löffler et al. (2024) in order to investigate how well a particular algorithm can recover a pre‐specified true value. We simulated shifts in latency between experimental conditions similar to Kiesel et al. (2008) to establish a known latency shift that must then be recovered empirically. We then evaluated how well peak latency, fractional area latency, and template matching algorithms recover those true simulated latency differences.

In this exploratory study, we hope to show that a template matching algorithm using the grand average as a variable template can successfully extract subject‐level P3 component latencies. Ideally, this algorithm improves psychometric properties in comparison to prior algorithms, shows high correlations with manually extracted data, accurately recovers simulated latency shifts, and presents an objective and efficient way to extract ERP latencies.

## 2 The Template Matching Algorithm

Broadly, the algorithm can be divided into four steps: generation of the dynamic template, application of similarity weights, optimization, and recovery of latency from free parameters.

### 2.1 Template Generation

We use the grand average ERP over all participants as a template and introduce two free parameters to allow the template to match individual differences in amplitude and latency. Both parameters lead to linear transformations of the template. The first parameter a scales the entire template and adjusts for individual differences in amplitude. The second parameter b stretches or compresses the entire template and adjusts for individual differences in time course.

**FIGURE 2:**
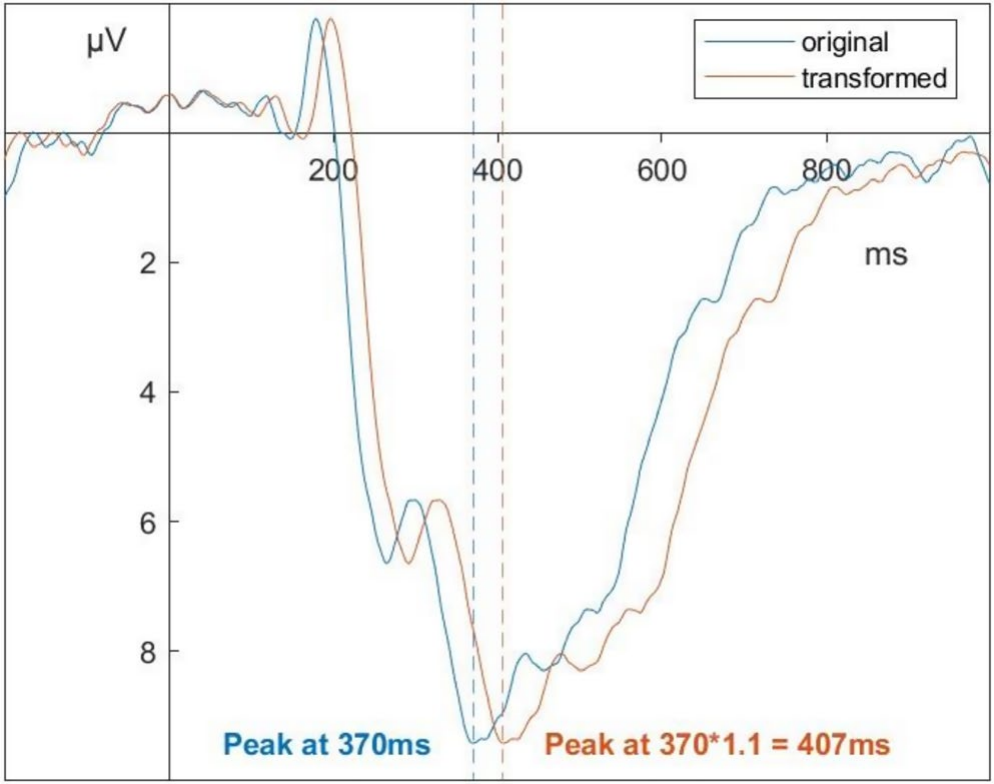
Transforming the latency of a signal using the stretching parameter b. Transformation of a signal is achieved by “stretching” the signal along the x‐Axis and spline‐interpolating the new values.

Implementation of the parameter a is straightforward: the entire template signal is multiplied by a. This results in linear transformations of the template along the amplitude axis. To implement the parameter b that enables linear transformations along the time axis, we simply stretch or compress the signal along the x‐axis. We then use spline interpolation to estimate the signal for a given transformed time‐point. For example, if the template has a peak signal strength of 9.4μV at 370 ms and the transformation parameter b=1.1, the transformed template will have the signal strength of 9.4μV at 370×1.1 ms. The signal strength at 370 ms will be equal to the spline interpolated signal strength of the untransformed template at $\frac{370}{1.1}$ ms (see Figure 2).

The algorithm allows these two free parameters to transform the template along the time‐ and amplitude–dimension and then optimizes the parameters for maximum similarity between the transformed template and subject‐level ERP.

### 2.2 Similarity Measures

We tested two different similarity measures in this paper. The first approach, referred to as the MINSQ method, minimizes the sum of squared distances between the transformed template $t_{i}\left(a,b\right)$ and the signal $s_{i}$ at all time points *i* weighted by a weighting vector $\omega_{i}$.

$$\underset{a,b}{\text{argmin}}=\sum_{i}\omega_{i}{\left(t_{i}\left(a,b\right)-s_{i}\right)}^{2}.$$

We tested several weighting vectors $\omega_{i}$. See the following section for additional details.

The second approach, referred to as the MAXCOR method, aims to maximize the (weighted) correlation between the transformed template $t_{i}\left(a,b\right)$ and the signal $s_{i}$ at all time points *i*, weighted by a weighting vector $\omega_{i}$. The optimization problem is defined as:

$$\underset{a,b}{\text{argmax}}=\frac{\mathrm{cov}\left(t\left(a,b\right),s;\omega \right)}{\sqrt{\mathrm{cov}\left(t\left(a,b\right),t\left(a,b\right);\omega \right)\mathrm{cov}\left(s,s;\omega \right)}},$$

where the weighted covariance is given by

$$\mathrm{cov}\left(t\left(a,b\right),s;\omega \right)=\frac{\sum_{i}\omega_{i}\cdot \left(t_{i}\left(a,b\right)-m\left(t\left(a,b\right);\omega \right)\right)\left(s_{i}-m\left(s;\omega \right)\right)}{\sum_{i}\omega_{i}},$$

and the weighted mean $m\left(t\left(a,b\right);\omega \right)$ by:

$$m\left(t\left(a,b\right);\omega \right)=\frac{\sum_{i}\omega_{i}t_{i}\left(a,b\right)}{\sum_{i}\omega_{i}}.$$

Since the parameter a scales the template $t_{i}\left(a,b\right)$ linearly along the amplitude‐axis, and the MAXCOR method relies on a weighted correlation that is invariant to linear transformations of amplitude, this method is not sensitive to changes in a. Therefore, for the MAXCOR method, we set a=1 and optimized only *b*.

### 2.3 Weighting Vectors

In order to impart weights on the matching procedure that allow placing more importance on those parts of the signal where the component of interest occurs, we use a weighting vector ω. Weighting windows indicate when the component of interest occurs.

We tested three different weighting windows in order to investigate which size leads to the best template matching results. We implemented the two measurement windows used in Sadus et al. (2024) in order to compare our results to theirs. The first window has a range from 250 to 700 ms and was applied to the fractional area latency algorithm, and the second window has a range from 250 to 900 ms and was applied to the peak latency algorithm. In order to include some of the activity of earlier components, we also tested a weighting window from 200 to 700 ms. Additionally, we evaluated a restrictive weighting window focusing on the peak of the P3 in the grand average from 300 to 600 ms.

Weighting windows are then combined with a weighting function to generate the weighting vector used in the template matching algorithm. A simple function to generate this weighting vector would be to assign 0 to all values outside of the window and 1 to all values inside of that window. This rectangular window would correspond to a Tukey window with α = 0 (Bloomfield 2004) and is also referred to as a Dirichlet window. The rectangular shape of this weighting window suggests a sudden drop in the relevance of a mismatch just at the weighting window's borders. However, such a sudden cut‐off is difficult to justify. Therefore, we tested two versions of weighting functions that include tapered edges.

**FIGURE 3:**
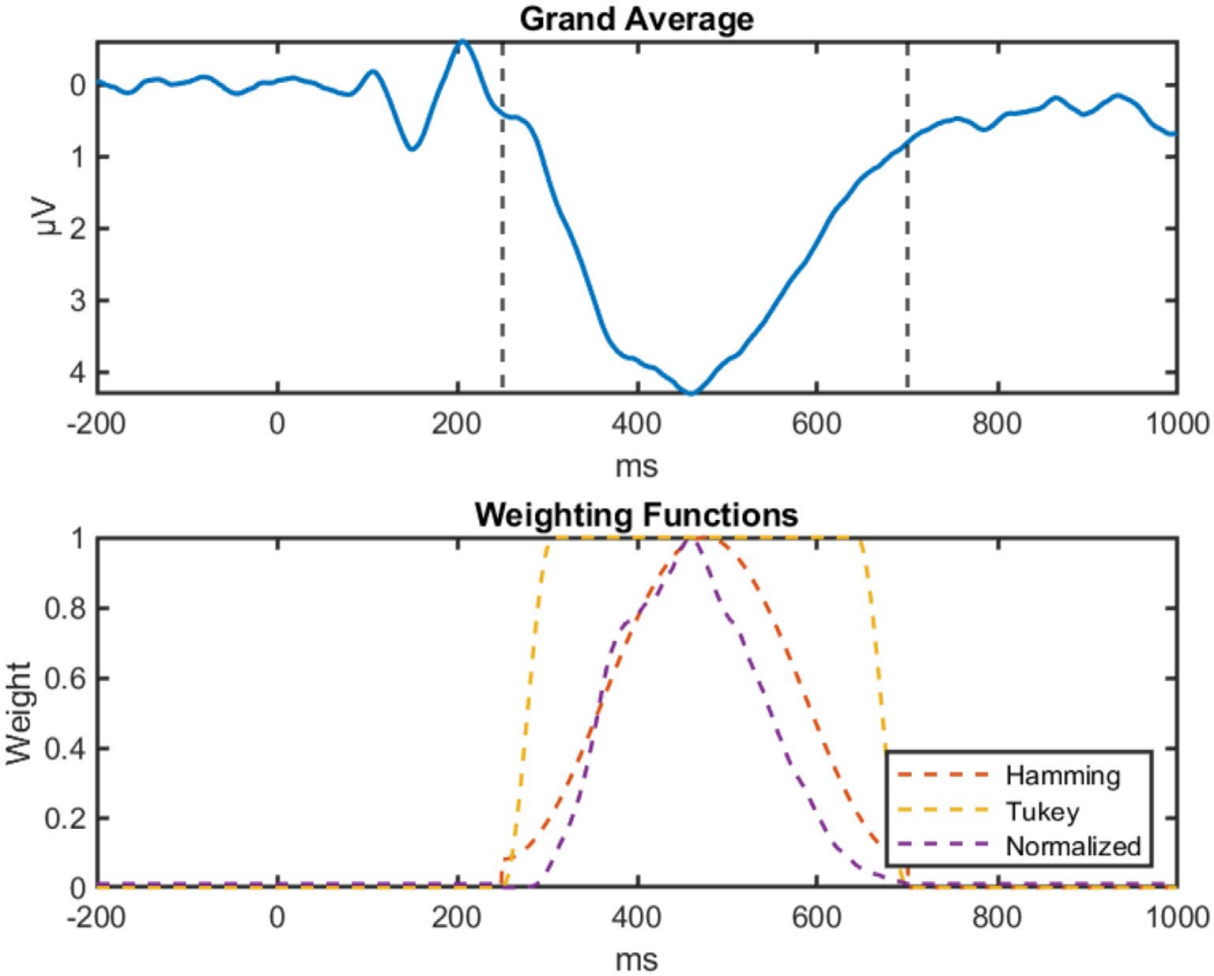
Overview of weighting functions. Weights are normalized to 1 for illustrative purposes. The illustration is based on a weighting window of 250 to 700 ms. Hamming refers to the use of a Hamming function to generate the weights. Tukey employs a Tukey window with α=0.25. Normalized refers to a weighting function that assigns weights based on the maximum‐normalized amplitude of the grand average. No weighting function would simply assign all time‐points a weight of 1.

We tested a Tukey window with α=0.25 and the Hamming window to slowly raise the weights from 0 outside of the measurement window to 1 inside the measurement window (Bloomfield 2004). Additionally, we also tested a weighting function that assigns weights based on the maximum‐normalized amplitude of the grand average. Here the difference between transformed template and signal is of maximum importance at the peak of the component in the grand average and decreases in importance at lower amplitudes. See Figure 3 for an overview of the different weighting functions. Lastly, we also evaluated the template matching approach without a weighting window, simply assigning all time points a weight of one regardless of the weighting window.

These weighting windows are defined based on the location of the component of interest in the grand average. In order to simplify the optimization, we chose to rescale the subject‐level ERP and fit it to the static grand average. Therefore, the actual computational implementation runs exactly inverse to the argument outlined here. This allows us to keep the weighting windows and weighting vectors constant and only transform the subject‐level ERP signal. It has no impact on the fit statistic or the recovered latency.

### 2.4 Penalty

In testing, we observed that the algorithms would occasionally converge on extreme values of b when no clear solution could be found. To mitigate this issue, we introduced a penalty function to the MAXCOR and MINSQ optimization functions. This penalty discourages any transformation where the signal latency deviates from the grand average by more than 50% (i.e., $b\leq 0.\bar{6}$ or b≥1.5) by multiplying the value of the objective function by $e^{b}$ if b≥1, or by $e^{\frac{1}{b}}$ if b<1. This ensures that large deviations from the grand average must show significant improvements in the optimization criteria should the algorithm converge on them. We intentionally only applied this penalty to $b\leq 0.\bar{6}$ or b≥1.5 in order not to bias plausible values of *b* towards 1. We tested all combinations of similarity measures, weighting windows, and weighting functions both with and without this penalty to ensure robustness and to observe the penalty's impact on convergence.

### 2.5 Optimization

All combinations of similarity measures, weighting windows, weighting functions, and penalty functions optimize the similarity measure using on the GlobalSearch algorithm (Ugray et al. 2007) implemented in the Global Optimization Toolbox for MATLAB (The Math Works, Inc 2024). This optimization algorithm uses a multistart heuristic approach to find the global minimum of a nonlinear differentiable function by evaluating multiple starting points for local minima. The number of starting points varies across optimization problems and is chosen by the algorithm to optimally explore the parameter space.

We constrained the parameter a with lower and upper bounds of $\left[0.2,20\right]$ and the parameter b with bounds of $\left[0.3,2\right]$. For the parameter a, we set the lower bound at 0.2 to avoid convergence near zero in cases where the signal is inconclusive and a flat line would fit the signal best. We opted for an upper bound of 20 as a generous and liberal limit that worked well during testing. We selected 0.3 as the lower bound for b because it seemed highly unlikely that an individual's latency would differ from the grand average latency by more than a factor of three. However, due to how our implementation compresses the signal when b increases, we encountered an issue where b>2 led to over 50% missing entries in the transformed template time series. To address this, we chose 2 as the upper bound and 0.3 as the lower bound, establishing a more conservative yet practical range. These bounds are intentionally asymmetrical, reflecting the practical constraints of our implementation and the need to balance realism with the algorithm's performance. During testing, these chosen bounds proved effective, helping to ensure that the algorithm did not converge on extreme or unrealistic values.

The algorithm returns both the optimal parameters as well as the resulting optimal similarity measure.

### 2.6 Fit

The optimal similarity measure returned by the algorithm can be used as an indicator of certainty. Very close fits of transformed template to subject‐level ERPs suggest a high degree of certainty and low impact of noise. Poor similarity measures, like correlations r<0.3 suggest that even the optimal transformation of the component in the template does not closely resemble the component structure in the subject‐level ERP. Therefore, we used of this fit statistic to remove latency estimates where even the best fit shows poor similarity between template and signal. Because the (weighted) correlation is easier to interpret, we used the correlation as an indicator of fit for both the MAXCOR and MINSQ approach and removed those latency estimates with correlations r<0.3. We chose the cutoff of 0.3 based on our experience with the algorithms. Manual inspection revealed that most subject‐level ERPs that produce correlations this low show no identifiable P3 component.

### 2.7 Recovery of Component Latency

We can use the set of optimal transformation parameters to recover the latency of subject‐level ERPs. Prior to this, the latency of the component of interest in the grand average needs to be quantified. This can be done either manually or using one of the standard algorithms, like peak latency or fractional area latency. Due to the high signal‐to‐noise ratio of the grand average, applying these algorithms is much less problematic than in noisy subject‐level ERPs. In our analysis, we use the 50% area latency algorithm proposed by Liesefeld (2018) with the respective weighting window as a measurement window to determine the latency in the grand average.

The latency of the component in the grand average $l_{\mathrm{GA}}$ is then scaled by the optimal latency shift parameter of subject j to obtain the latency in the subject‐level ERP.

### 2.8 Other Extraction Methods

We defined peak latency as the time point of maximum positive voltage deflection within a fixed measurement window, where the deflection is larger than the average of the three neighboring points on both sides (Luck 2014).

We determined fractional area latency using the 50% area latency approach. All values of the signal below 0 μV were set to 0. Then we summed up the signal within the measurement window and extracted the two time‐points between which the cumulative signal exceeds 50% of the total. The exact latency was then determined through linear interpolation.

We also tested two modifications to fractional area latency suggested by Liesefeld (2018). First, we subtracted half of the maximum amplitude from the signal as a relative baseline (see also Wascher et al. 2022). Essentially, we set all values of the signal below 50% of the maximum amplitude to 0 (Liesefeld A). All other steps were identical to those in the standard 50% area latency algorithm.

Additionally, we employed the *liesefeld.m* function using 30% of the peak amplitude as a baseline and constraining the measurement window by the crossings of the baseline on both sides of the component peak (Liesefeld B).

## 3 Study 1

### 3.1 Method

The template matching algorithm is implemented in MATLAB (Version 2024a).^1^ All code necessary to reproduce this work be found online (https://github.com/SLesche/template‐matching‐paper). We conducted all data preprocessing and statistical analyses using R (Version 4.3.0; R Core Team 2022). We furthermore used the R‐packages *afex* (Version 1.3.1; Singmann et al. 2023), *emmeans* (Version 1.10.2; Lenth 2023), *flextable* (Version 0.9.6; Gohel and Skintzos 2023), *knitr* (Version 1.46; Xie 2015), *papaja* (Version 0.1.2; Aust and Barth 2022), *rmarkdown* (Version 2.26; Xie et al. 2018, 2020), and *tidyverse* (Version 2.0.0; Wickham et al. 2019).

All analyses presented here are based on data previously published in Löffler et al. (2024). The original data contain EEG recordings from 12 different tasks. We focused on the same three tasks that have been already analyzed by Sadus et al. (2024).

#### 3.1.1 Participants

The empirical evaluation of our algorithm that is presented in the first part of this paper follows Sadus et al. (2024). Their analyses used a sample of 30 young (18–21 years old, $M_{\mathrm{age}}$ = 19.37, $SD_{\mathrm{age}}$ = 0.76) and 30 old participants (50–60 years old, $M_{\mathrm{age}}$ = 55.83, $SD_{\mathrm{age}}$ = 2.87), representing the 30 youngest and 30 oldest participants from the overall study (Löffler et al. 2024).

All participants had normal or corrected‐to‐normal vision. None of the participants had neurological or mental disorders, used psychotropic drugs, wore a pacemaker, or suffered from color vision deficiency. All participants provided informed consent prior to participation and received 75€ or course credit for participation.

#### 3.1.2 Tasks

All participants completed a battery of 12 tasks including the three tasks we used for this manuscript: a Flanker Task, an Nback Task, and a Switching Task. Each assesses one of the three executive functions proposed by Miyake et al. (2000). Löffler et al. (2024) programmed all tasks using MATLAB (The Math Works, Inc 2022) and the software package Psychtoolbox (Version 3‐0.13) (Brainard and Vision 1997; Kleiner et al. 2007; Pelli and Vision 1997). We presented stimuli centrally against a black background and instructed participants to respond as quickly and accurately as possible.

##### 3.1.2.1 Flanker Task

We employed a standard Arrow Flanker task (Eriksen and Eriksen 1974) to evaluate a participant's *inhibition* ability. A central arrow was flanked by additional arrows appeared on the screen, with the flanking arrows either pointing in the same (congruent condition) or opposite direction (incongruent condition) to the central arrow. All participants had to identify the direction of the central arrow while ignoring the flanking arrows. Each participant completed 20 practice trials followed by 200 trials (100 for each condition).

##### 3.1.2.2 Nback Task

An adapted Nback task (Scharinger et al. 2015) assessed participants' *updating* abilities. During presentation of a stream of letters, participants had to indicate whether the current letter matched either a predefined target letter (0‐back condition) or the letter presented immediately before (1‐back condition). In the original study, participants also completed a 2‐back condition. Following Sadus et al. (2024), we excluded this condition from our analysis, due to an unclear and therefore unsuitable ERP structure. All participants completed 30 practice trials and 96 experimental trials per condition.

##### 3.1.2.3 Switching Task

We administered a Switching task (Sudevan and Taylor 1987) to measure participants' *shifting* ability. We presented colored digits ranging from 1 to 9. Participants had to categorize them based on predefined rules signaled by the colored fixation cross before each trial and the colored digit following the fixation cross. They had to either indicate whether the digit was greater than or less than 5 or whether the digit was odd or even. Depending on the color, participants either maintained the same rule as the previous trial or switched to the alternate rule. Each participant completed 30 practice trials followed by 384 experimental trials.

#### 3.1.3 Procedure

The original study consisted of three test sessions. The three tasks analyzed here were all conducted in the first session. The original study included two additional measurement occasions which we will not report here.

#### 3.1.4 EEG Recording and Processing

We continuously recorded EEG data using 32 equidistant Ag/AgCl electrodes, the BrainAmp amplifier system, and the BrainVision recorder, and took additional electrooculogram (EOG) measures with two electrodes placed above and below the left eye to correct for ocular artifacts. We maintained all impedances below 5 kΩ. We recorded the signal with a sampling rate of 1000 Hz, an online bandpass filter of 0.1–1000 Hz and referenced the signal online to Cz.

During preprocessing, we removed artifacts using ICA on a cloned dataset down‐sampled to 200 Hz and filtered with a high‐pass filter of 1 Hz. We further removed line noise using the CleanLine function (Mullen 2012). We detected bad channels using a *z*‐value threshold of 3.29 as per the EPOS pipeline (Rodrigues et al. 2021) and interpolated channels that were removed following this procedure using the spherical spline interpolation procedure implemented in EEGlab. Then, we re‐referenced the data using an average referencing scheme. Based on a threshold of 1000μV and excluding data more than 5 SDs away from the mean, we automatically detected and removed a maximum of 5% of segments per iteration containing artifacts in the ICA dataset. We used the InfoMax algorithm in the ICA and applied the resulting decomposition to the original dataset. We removed ICs determined to be less than 50% likely to be brain activity by the ICLabel Algorithm (Pion‐Tonachini et al. 2019). Then, we low‐pass filtered the data using a Butterworth filter with varying cutoff frequencies (4, 8, 16, 32 Hz with a roll‐off of 12 dB/octave, and no filter) and segmented it into 1200 ms epochs starting 200 ms prior to stimulus onset. Again, we automatically detected and removed segments containing artifacts. Finally, we baseline corrected segments using the 200 ms pre‐stimulus period. We conducted ERP analyses in MATLAB (Version 2024a; The Math Works, Inc 2024) using the EEGLAB software (Delorme and Makeig 2004). We only included correct trials in the analysis and investigated the P3 at the Pz electrode in accordance with existing literature (Polich 2012; Verleger 2020). On average, we retained 85 trials in the congruent condition and 84 trials for the incongruent condition of the flanker task, 82 trials in the 0‐back and 78 trials in the 1‐back condition of the Nback task, and 149 trials in both the switch and repeat condition of the switching task.

#### 3.1.5 Latency Extraction Techniques

**FIGURE 4:**
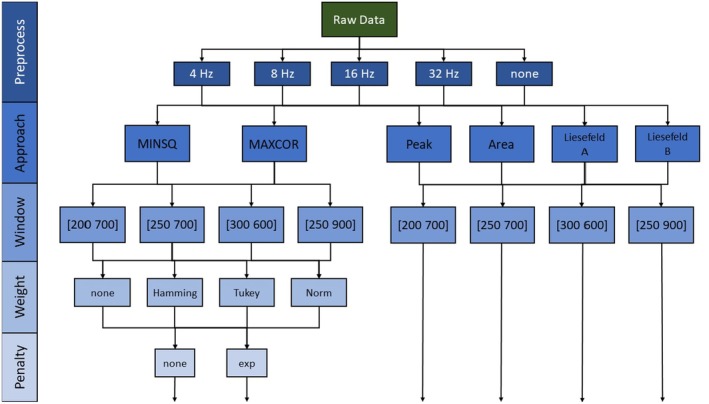
Overview of extraction algorithms. Data were preprocessed with five different filter settings noted here in Hz. The approaches refer to the distance‐based template matching algorithm (MINSQ), the correlation‐based template matching algorithm (MAXCOR), traditional peak latency (Peak), traditional fractional area latency (Area), a modified area latency approach adjusting the baseline to 50% of the peak amplitude (Liesefeld A), and a modified latency approach adjusting the baseline and constraining the measurement window by the onset and offset of the component (Liesefeld B). The windows refer to the lower and upper bound (in ms) of the measurement windows for the component. Weight refers to the different weighting functions used in the template matching approach. Penalty denotes the inclusion of a penalty of extreme transformation parameters.

We compared all versions of a template matching algorithm resulting from the combinations of similarity measures, weighting windows, weighting functions, and penalty methods to traditional approaches such as peak latency, fractional area latency, and the two modified fractional area latency algorithms proposed by Liesefeld (2018)—Liesefeld A and Liesefeld B. For peak latency and 50% area latency algorithms, we tried all weighting windows as measurement windows but focus on the results using the respective measurement windows used by Sadus et al. (2024). Here, the peak latency algorithm is used with a window of 250–900 ms and the area latency algorithms with a window of 250–700 ms. See Figure 4 for an overview.

#### 3.1.6 Empirical Evaluation

In order to compare our newly proposed algorithm to existing extraction methods, we applied all versions of the template matching algorithm as well as the peak latency algorithm, the 50% area latency algorithm, and the two modified area latency algorithms (Liesefeld 2018; Wascher et al. 2022). In order to evaluate the different algorithms, we compared the reliability and validity of the subject latencies they extract. Specifically, we computed two‐part coefficient alpha to estimate reliability and the intra‐class correlation of latencies extracted by an algorithm compared to an expert ERP researcher focusing on absolute agreement. For this purpose, we used the manually extracted latencies from Sadus et al. (2024). An optimal extraction algorithm should extract reliable latency estimates and show high correlations with manual extraction.

To estimate the uncertainty due to sampling variability, we computed bootstrapped 95% confidence intervals for the mean algorithm performance. Specifically, we resampled participants with replacement 5000 times and recomputed all statistics for each resample. The confidence intervals correspond to the 2.5th and 97.5th percentiles of the resulting bootstrap distribution of mean performance.

### 3.2 Results

**FIGURE 5:**
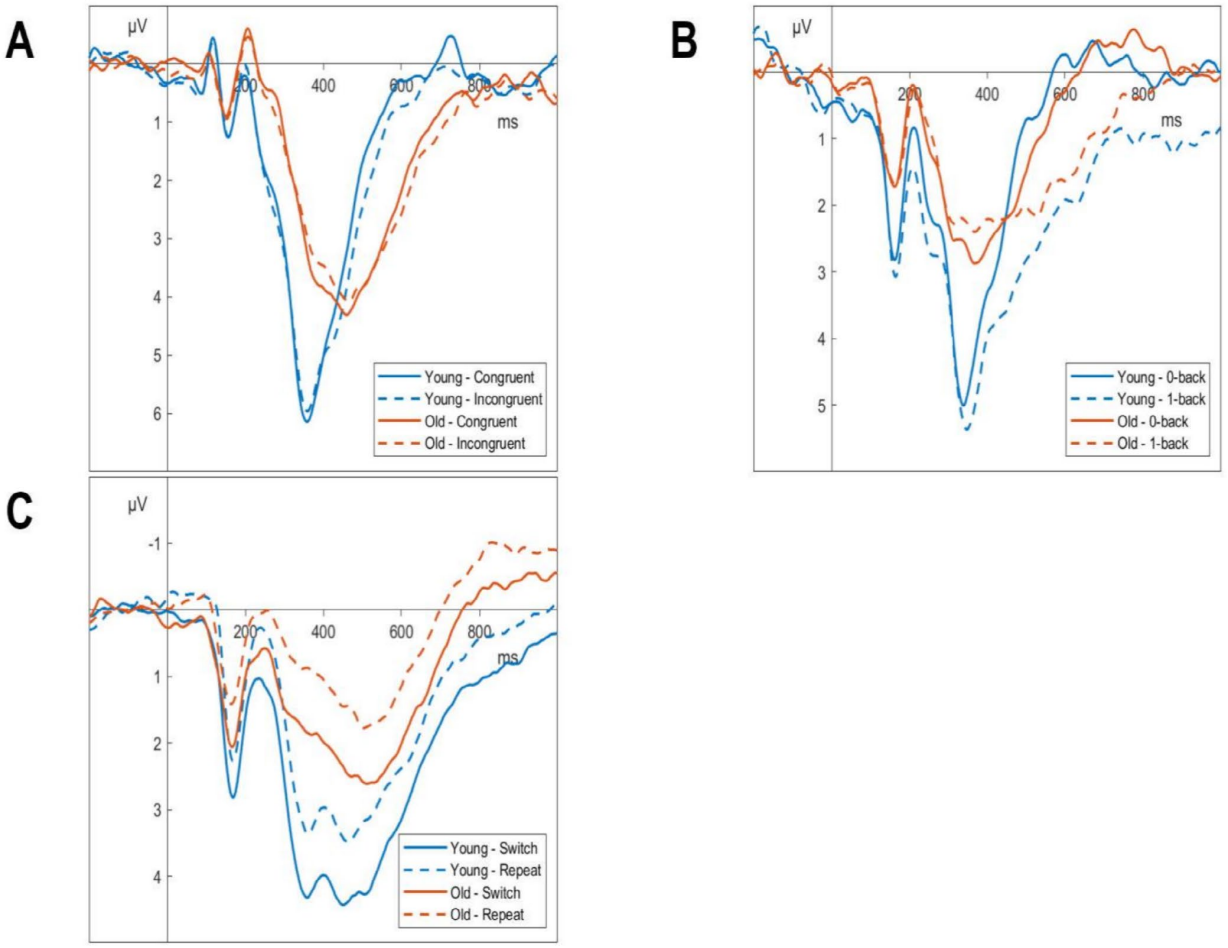
Grand averages of the tasks used at Pz with a 32 Hz low‐pass filter. (A) Grand Average of the Flanker Task, split by condition and age group. (B) Grand Average of the Nback Task, split by condition and age group. (C) Grand Average of the Switching Task, split by condition and age group.

The grand averages of the three tasks in Study 1 and the data containing the full set of participants are displayed in Figure 5.

In the template matching algorithms, we removed all latencies where the fit statistic was below 0.3. Additionally, we removed latency estimates deviating more than 3 standard deviations from the task and condition‐specific mean for all algorithms. We will focus our analysis on the mean values across tasks and preprocessing pipelines because a broadly applicable algorithm should show good average performance across a range of tasks. Quality estimates split by task and preprocessing step can be found in the tables in Tables A1–A6. An overview of the distribution of the fit statistic can be found in Figure A1.

#### 3.2.1 Descriptive Statistics

The percentage of missing values varied systematically depending on the extraction algorithm (see Table 1). Across tasks, preprocessing steps, weighting windows, weighting functions, and without a penalty the MINSQ algorithm resulted in 20.33% missing values and the MAXCOR algorithm in 13.24% missing values. Penalizing more extreme transformation parameters reduced the percentage of missing values to 6.38% for the MINSQ algorithm and 4.21% for the MAXCOR algorithm. The peak latency algorithm resulted in 1.78% missing values, with 2.72% for the fractional area latency algorithm, and 3.61% and 0.89% missing values for the modified area latency algorithms using either the maximum peak amplitude (Liesefeld A) or constraining the measurement window by the on‐ and offset of the component (Liesefeld B), respectively. For reference, the expert ERP researcher in Sadus et al. (2024) classified 4.05% of individual ERPs as missing values.

**TABLE 1:** Missing values for different algorithms: empirical evaluation.

Approach	Weight	[200 700]		[250 700]		[250 900]		[300 600]	
		Penalized	None	Penalized	None	Penalized	None	Penalized	None
MAXCOR	None	5.83	9.11	5.83	9.11	5.83	9.11	5.83	9.11
	Hamming	3.67	16.50	3.11	17.56	4.22	9.22	5.11	25.94
	Tukey	4.39	16.06	3.89	15.89	4.83	8.67	4.67	24.39
	Normalized	2.50	10.61	2.39	10.33	2.17	11.28	3.06	8.94
MINSQ	None	6.67	21.17	6.67	21.17	6.67	21.17	6.67	21.17
	Hamming	5.72	19.55	4.83	19.50	9.56	26.72	8.17	21.22
	Tukey	8.11	21.89	6.89	21.50	9.67	25.72	7.50	21.17
	Normalized	3.50	15.94	3.61	15.78	3.61	17.28	4.22	14.33
Peak	None		1.22		1.22		1.78		1.28
Area	None		2.61		2.72		3.44		2.39
Liesefeld A	None		3.50		3.61		3.83		2.78
Liesefeld B	None		0.89		0.89		1.39		0.67

*Note:* Percent of missing values per algorithm. The rows indicate combinations of similarity measures and weighting functions. The columns denote the measurement window and indicate if a penalty was used. MAXCOR and MINSQ refer to the template matching algorithms maximizing the correlation or minimizing the squared distance, respectively. Peak refers to the peak latency approach, area to a standard 50% fractional area latency approach. Liesefeld A and Liesefeld B refer to modified fractional area latency approaches proposed by Liesefeld et al. (2016, 2018). Liesefeld A uses 50% of the peak amplitude as the new baseline. Liesefeld B uses 30% of the peak amplitude as the baseline and additionally constrains the measurement window by the on‐ and offset of the component.

#### 3.2.2 Reliability

**FIGURE 6:**
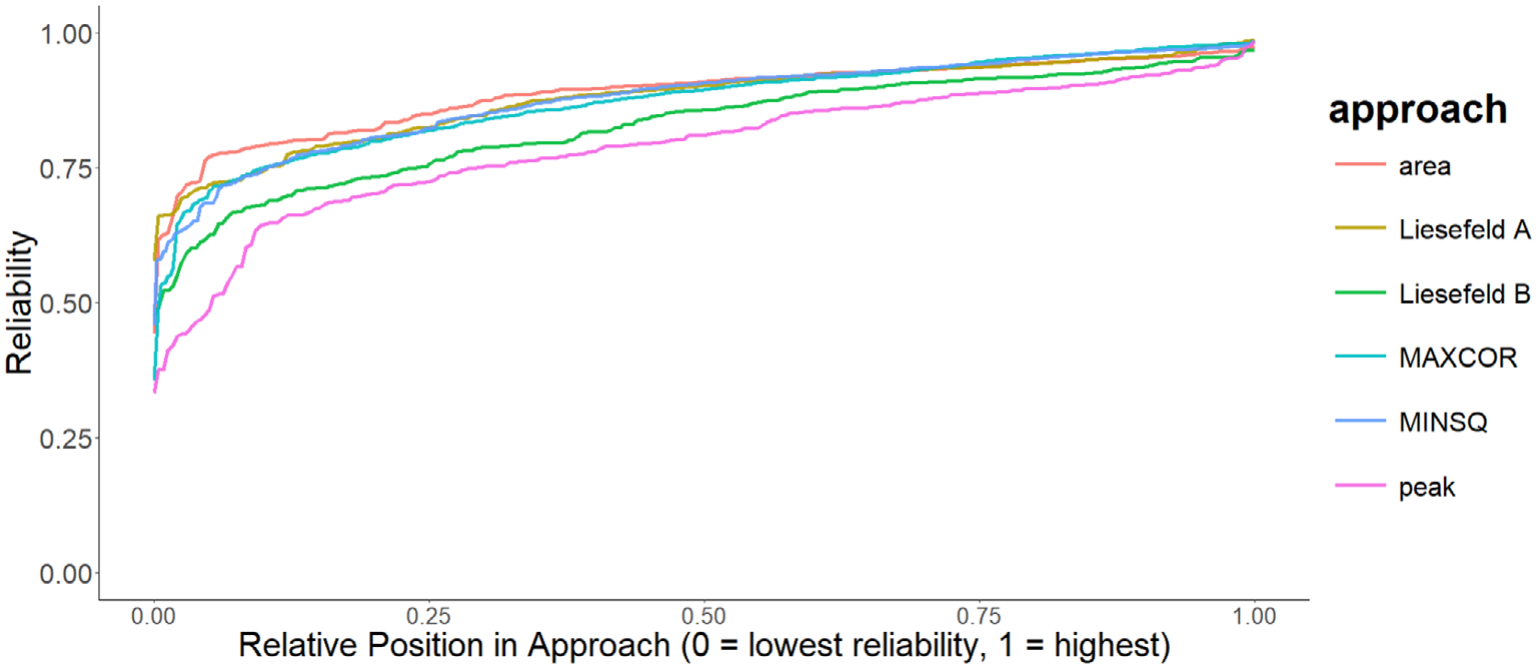
Reliability estimates by extraction method. Overview of reliability estimated using two‐part coefficient alpha by latency extraction method. The plot shows all reliability estimates across tasks, preprocessing settings and weighting windows ordered by size within a given approach. For the template matching approaches, only the normalized weighting function is displayed. MAXCOR and MINSQ refer to the template matching algorithms maximizing the correlation or minimizing the squared distance, respectively. Peak refers to the peak latency approach, area to a standard 50% fractional area latency approach. Liesefeld A and Liesefeld B refer to modified fractional area latency approaches proposed by Liesefeld et al. (2016, 2018). Liesefeld A uses 50% of the peak amplitude as the new baseline. Liesefeld B uses 30% of the peak amplitude as the baseline and additionally constrains the measurement window by the on‐ and offset of the component.

The reliability coefficients estimated using two‐part coefficient alpha are reported in Table 2 and the full distribution is shown in Figure 6. Bootstrapped 95% confidence intervals are provided in brackets following the point estimates. Across tasks and preprocessing steps, the 50% area latency algorithm with the window (250–700 ms) used in Sadus et al. (2024) resulted in the highest average reliability (α $=0.90,95\%\mathrm{CI}\;\left[0.83,0.94\right]$). Across tasks, preprocessing steps, weighting windows, weighting functions, and penalty settings, the MINSQ algorithm showed a mean reliability of $\alpha =0.87,95\%\mathrm{CI}\;\left[0.79,0.90\right]$ and the MAXCOR algorithm a mean reliability of α $=0.86,95\%\mathrm{CI}\;\left[0.80,0.89\right]$. The best reliability for the template matching algorithms was achieved when using the MINSQ algorithm with normalized weights and an exponential penalty (α $=0.89,95\%\mathrm{CI}\;\left[0.82,0.92\right]$). The modified fractional area latency algorithm using the 50% maximum amplitude (Liesefeld A) resulted in an average reliability of $\alpha =0.89,95\%\mathrm{CI}\;\left[0.83,0.92\right]$, the modified algorithm constraining the measurement window by the on‐ and offset of the component (Liesefeld B) resulted in an average reliability of α $=0.84,95\%\mathrm{CI}\;\left[0.76,0.88\right]$ and the peak latency algorithm in an average reliability of $\alpha =0.78,95\%\mathrm{CI}\;\left[0.70,0.83\right]$. For reference, manual extraction by an expert ERP researcher in Sadus et al. (2024) yielded average reliability estimates of $r_{\mathrm{tt}}=0.89$.

**TABLE 2:** Reliability for different algorithms: empirical evaluation.

Approach	Weight	[200 700]		[250 700]		[250 900]		[300 600]	
		Penalized	None	Penalized	None	Penalized	None	Penalized	None
MAXCOR	None	0.88 [0.82, 0.91]	0.88 [0.83, 0.91]	0.88 [0.82, 0.91]	0.88 [0.83, 0.91]	0.88 [0.82, 0.91]	0.88 [0.83, 0.91]	0.88 [0.82, 0.91]	0.88 [0.83, 0.91]
	Hamming	0.89 [0.83, 0.92]	0.91 [0.85, 0.94]	0.84 [0.76, 0.89]	0.84 [0.69, 0.89]	0.86 [0.76, 0.91]	0.84 [0.72, 0.89]	0.81 [0.72, 0.85]	0.78 [0.63, 0.84]
	Tukey	0.90 [0.85, 0.92]	0.90 [0.85, 0.92]	0.86 [0.78, 0.91]	0.85 [0.74, 0.91]	0.86 [0.76, 0.91]	0.83 [0.72, 0.89]	0.81 [0.72, 0.86]	0.81 [0.69, 0.87]
	Normalized	0.87 [0.80, 0.91]	0.87 [0.80, 0.91]	0.87 [0.79, 0.90]	0.88 [0.80, 0.91]	0.88 [0.81, 0.91]	0.87 [0.80, 0.91]	0.87 [0.80, 0.91]	0.85 [0.75, 0.9]
MINSQ	None	0.88 [0.81, 0.91]	0.88 [0.81, 0.92]	0.88 [0.81, 0.91]	0.88 [0.81, 0.92]	0.88 [0.81, 0.91]	0.88 [0.81, 0.92]	0.88 [0.81, 0.91]	0.88 [0.81, 0.92]
	Hamming	0.89 [0.80, 0.93]	0.86 [0.71, 0.93]	0.89 [0.81, 0.93]	0.84 [0.71, 0.91]	0.86 [0.78, 0.89]	0.8 [0.65, 0.86]	0.86 [0.76, 0.92]	0.84 [0.69, 0.90]
	Tukey	0.89 [0.84, 0.92]	0.89 [0.82, 0.93]	0.88 [0.79, 0.92]	0.86 [0.73, 0.92]	0.86 [0.78, 0.89]	0.81 [0.65, 0.87]	0.87 [0.78, 0.92]	0.85 [0.70, 0.92]
	Normalized	0.89 [0.82, 0.92]	0.87 [0.75, 0.91]	0.89 [0.82, 0.92]	0.86 [0.76, 0.91]	0.89 [0.83, 0.92]	0.87 [0.76, 0.91]	0.87 [0.8, 0.91]	0.86 [0.76, 0.90]
Peak	None		0.8 [0.69, 0.85]		0.81 [0.72, 0.85]		0.78 [0.69, 0.83]		0.77 [0.66, 0.82]
Area	None		0.90 [0.83, 0.93]		0.90 [0.83, 0.94]		0.86 [0.79, 0.90]		0.89 [0.79, 0.93]
Liesefeld A	None		0.88 [0.82, 0.92]		0.89 [0.83, 0.92]		0.85 [0.79, 0.90]		0.88 [0.82, 0.92]
Liesefeld B	None		0.83 [0.74, 0.88]		0.84 [0.76, 0.88]		0.82 [0.74, 0.86]		0.83 [0.75, 0.87]

*Note:* Reliability estimated using two‐part coefficient alpha by latency extraction method. Values in brackets provide bootstrapped 95% confidence intervals. The rows indicate combinations of similarity measure and weighting function. The columns denote the measurement window and indicate if a penalty was used. MAXCOR and MINSQ refer to the template matching algorithms maximizing the correlation or minimizing the squared distance, respectively. Peak refers to the peak latency approach, area to a standard 50% fractional area latency approach. Liesefeld A and Liesefeld B refer to modified fractional area latency approaches proposed by Liesefeld et al. (2016, 2018). Liesefeld A uses 50% of the peak amplitude as the new baseline. Liesefeld B uses 30% of the peak amplitude as the baseline and additionally constrains the measurement window by the on‐ and offset of the component.

#### 3.2.3 Agreement With Manual Extraction

**FIGURE 7:**
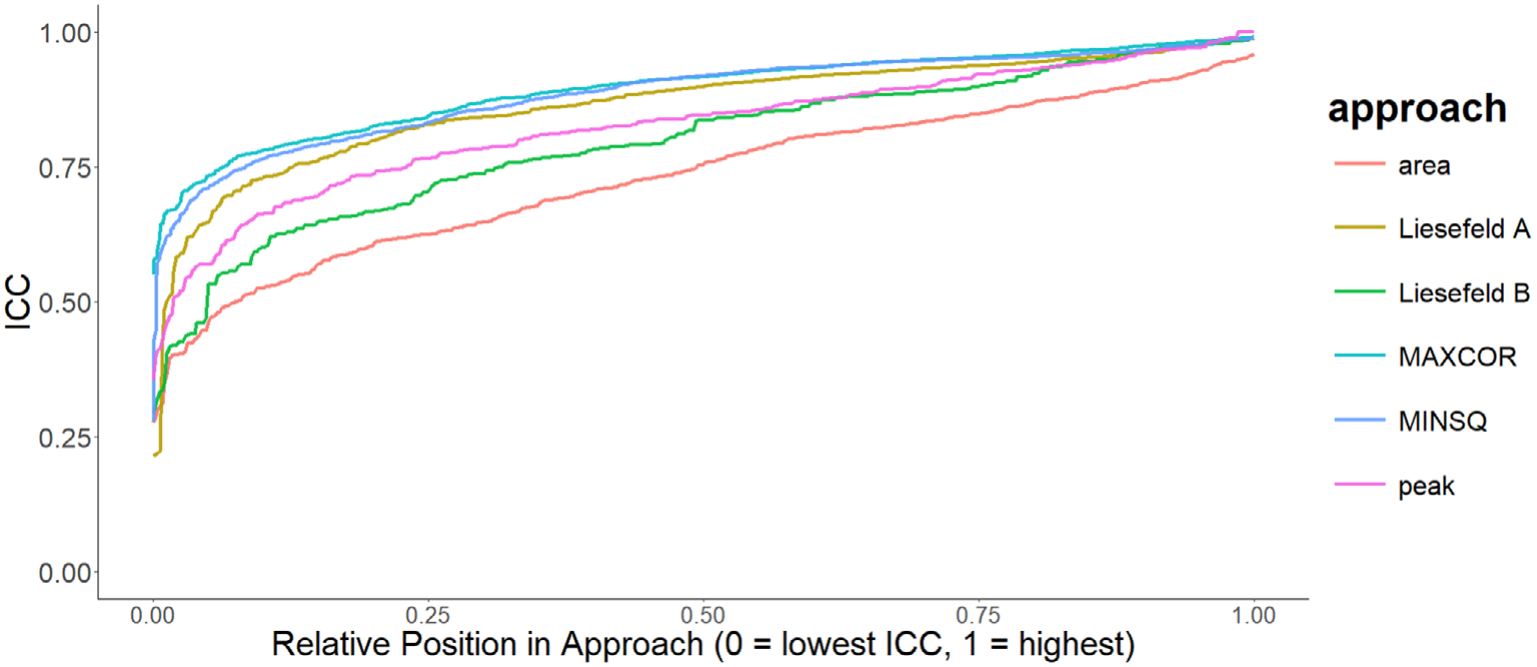
ICC estimates by extraction method. Overview of ICC focusing on absolute agreement between automated approaches and the expert ERP researcher from Sadus et al. (2024) by latency extraction method. The plot shows all ICC estimates across tasks, preprocessing settings and weighting windows ordered by size within a given approach. For the template matching approaches, only the normalized weighting function is displayed. MAXCOR and MINSQ refer to the template matching algorithms maximizing the correlation or minimizing the squared distance, respectively. Peak refers to the peak latency approach, area to a standard 50% fractional area latency approach. Liesefeld A and Liesefeld B refer to modified fractional area latency approaches proposed by Liesefeld et al. (2016, 2018). Liesefeld A uses 50% of the peak amplitude as the new baseline. Liesefeld B uses 30% of the peak amplitude as the baseline and additionally constrains the measurement window by the on‐ and offset of the component.

Intra‐class correlations focusing on absolute agreement, as implemented in the R package *irr*, between automatically extracted ERP latencies and those extracted by Sadus et al. (2024) can be found in Table 3 and the full distribution is shown in Figure 7. The choice of weighting function had considerable impact on the intra‐class correlation (ICC). Hamming and Tukey weighting functions showed a mean $\mathrm{ICC}=0.79,95\%\mathrm{CI}\;\left[0.72,\;0.83\right]$ across tasks, preprocessing steps, weighting windows, and template matching algorithms. The grand average normalized weighting functions showed a mean $\mathrm{ICC}=0.89,95\%\mathrm{CI}\;\left[0.84,\;0.91\right]$.

**TABLE 3:** ICC for different algorithms: empirical evaluation.

Approach	Weight	[200 700]		[250 700]		[250 900]		[300 600]	
		Penalized	None	Penalized	None	Penalized	None	Penalized	None
MAXCOR	None	0.80 [0.72, 0.85]	0.80 [0.71, 0.85]	0.80 [0.72, 0.85]	0.80 [0.71, 0.85]	0.80 [0.72, 0.85]	0.80 [0.71, 0.85]	0.80 [0.72, 0.85]	0.80 [0.71, 0.85]
	Hamming	0.87 [0.80, 0.91]	0.85 [0.79, 0.89]	0.83 [0.75, 0.89]	0.81 [0.73, 0.88]	0.8 [0.71, 0.86]	0.79 [0.7, 0.85]	0.72 [0.63, 0.77]	0.70 [0.62, 0.76]
	Tukey	0.86 [0.79, 0.90]	0.84 [0.77, 0.88]	0.82 [0.75, 0.88]	0.79 [0.69, 0.88]	0.81 [0.73, 0.86]	0.79 [0.71, 0.85]	0.71 [0.63, 0.78]	0.69 [0.62, 0.75]
	Normalized	0.89 [0.85, 0.92]	0.90 [0.84, 0.92]	0.90 [0.86, 0.93]	0.90 [0.85, 0.93]	0.89 [0.85, 0.92]	0.88 [0.82, 0.91]	0.89 [0.84, 0.92]	0.89 [0.84, 0.92]
MINSQ	None	0.74 [0.63, 0.81]	0.78 [0.69, 0.83]	0.74 [0.63, 0.81]	0.78 [0.69, 0.83]	0.74 [0.63, 0.81]	0.78 [0.69, 0.83]	0.74 [0.63, 0.81]	0.78 [0.69, 0.83]
	Hamming	0.86 [0.79, 0.89]	0.84 [0.77, 0.88]	0.85 [0.78, 0.89]	0.82 [0.74, 0.87]	0.70 [0.59, 0.77]	0.69 [0.59, 0.75]	0.81 [0.72, 0.86]	0.75 [0.67, 0.81]
	Tukey	0.83 [0.77, 0.87]	0.82 [0.75, 0.86]	0.84 [0.76, 0.88]	0.83 [0.75, 0.87]	0.75 [0.64, 0.81]	0.72 [0.63, 0.80]	0.79 [0.69, 0.86]	0.77 [0.67, 0.84]
	Normalized	0.89 [0.85, 0.92]	0.89 [0.84, 0.91]	0.90 [0.85, 0.92]	0.89 [0.84, 0.91]	0.89 [0.84, 0.92]	0.88 [0.82, 0.90]	0.88 [0.83, 0.92]	0.86 [0.79, 0.90]
Peak	None		0.83 [0.75, 0.87]		0.85 [0.78, 0.89]		0.83 [0.76, 0.88]		0.78 [0.71, 0.83]
Area	None		0.80 [0.72, 0.85]		0.78 [0.69, 0.84]		0.70 [0.6, 0.79]		0.65 [0.54, 0.72]
Liesefeld A	None		0.88 [0.82, 0.92]		0.90 [0.85, 0.93]		0.84 [0.77, 0.91]		0.83 [0.77, 0.87]
Liesefeld B	None		0.78 [0.69, 0.87]		0.81 [0.72, 0.88]		0.79 [0.71, 0.88]		0.8 [0.72, 0.86]

*Note:* Intra‐class correlations focusing on absolute agreement. Values in brackets provide bootstrapped 95% confidence intervals. The rows indicate combinations of similarity measure and weighting function. The columns denote the measurement window and indicate if a penalty was used. MAXCOR and MINSQ refer to the template matching algorithms maximizing the correlation or minimizing the squared distance, respectively. Peak refers to the peak latency approach, area to a standard 50% fractional area latency approach. Liesefeld A and Liesefeld B refer to modified fractional area latency approaches proposed by Liesefeld et al. (2016, 2018). Liesefeld A uses 50% of the peak amplitude as the new baseline. Liesefeld B uses 30% of the peak amplitude as the baseline and additionally constrains the measurement window by the on‐ and offset of the component.

Using the MINSQ or MAXCOR algorithm in conjunction with normalized weights yielded mean ICCs of $\mathrm{ICC}=0.88;95\%\mathrm{CI}=\left[0.84,0.91\right]$ and ICC=0.90; $95\%\mathrm{CI}=\left[0.84,0.92\right]$, respectively. The peak latency algorithm showed a mean $\mathrm{ICC}=0.83;95\%\mathrm{CI}=\left[0.76,0.88\right]$, the 50% area latency algorithm showed a mean ICC=0.78; $95\%\mathrm{CI}=\left[0.69,0.84\right]$, the modified area latency algorithm using the maximum amplitude (Liesefeld A) showed a mean $\mathrm{ICC}=0.90;95\%\mathrm{CI}=\left[0.85,0.93\right]$, and the modified area latency algorithm (Liesefeld B) constraining the measurement window by the on‐ and offset of the component showed a mean $\mathrm{ICC}=0.81;95\%\;\mathrm{CI}=\left[0.72,\;0.88\right]$.

### 3.3 Discussion

Several versions of the new algorithm showed good reliability and high agreement with manual extraction by an expert ERP researcher. Specifically, template matching algorithms using the normalized weighting function showed high agreement with manual extraction.

The modification proposed by Liesefeld et al. (2016)—adjusting the baseline in the fractional area latency approach to 50% of the peak amplitude (Liesefeld A)—performed slightly better than template matching approaches when applying a measurement window from 250 to 700 ms. Here, Liesefeld A showed slightly higher reliability and similar average agreement to a manual researcher compared to the best template matching approaches.

However, the bootstrapped confidence intervals reveal substantial variability resulting from participant‐level resampling. The confidence intervals of the best template matching algorithms and the best traditional approaches overlap almost entirely. Small differences in the reliability and agreement are likely due to sampling variability and do not necessarily reflect true differences in algorithm performance. Nonetheless, descriptively, MINSQ, MAXCOR, and Liesefeld A appear to perform better than the other available algorithms.

When extending the view to all tested measurement windows in order to investigate the robustness of extraction algorithms, the MINSQ and MAXCOR approach using the normalized weighting function showed higher ICCs on average than other extraction algorithms. Notably, the largest performance gains are visible in the lowest 20% of ICCs. These “worst ICCs” are lower in traditional approaches than in the MINSQ and MAXCOR approach (see Figure 7). The worst performances of the template matching algorithms seem to be better than the worst performances of traditional approaches.

These results suggest that either the MINSQ or MAXCOR approach in combination with a normalized weighting function and a penalty provides a good measure of P3 latency as they accurately reflect the pipeline that provided the best performance in previous studies (Sadus et al. 2024; Schubert et al. 2023). The ICC of the template matching approaches is robust to the weighting window employed, yielding similar results for all window settings.

Importantly, even though expert researchers have been shown to provide the best measures of P3 latency (Sadus et al. 2024), they do not measure the true latency without measurement error. We therefore chose to expand our validation through empirical results by a simulation study that will allow us to investigate which latency extraction method can recover true latencies the best.

## 4 Study 2

We expanded the empirical validation by a simulation study based on real data in order to investigate how well a particular algorithm can recover a pre‐specified true value in a set of realistic ERPs. We opted to simulate shifts in latency between experimental conditions similar to Kiesel et al. (2008). This allowed us to simulate highly realistic ERPs and introduce a known true value of the experimental effect. We then evaluated how well each algorithm is able to reproduce this known experimental effect.

### 4.1 Method

All analyses presented here are based on data originally published in Löffler et al. (2024). The simulation is limited to the task and condition that yielded the grand average with the most typical P3—the congruent condition of the Flanker task. We included the complete set of *N* = 148 participants ($M_{\mathrm{age}}$ = 31.52, $SD_{\mathrm{age}}$ = 13.91). For detailed information regarding the Flanker task, see the method section of study 1 or Löffler et al. (2024). The procedure was identical to the procedure described in study 1 except that the simulation focuses on only the flanker task.

EEG preprocessing deviated only slightly from the protocol described in study 1. Instead of using the original recording frequency of 1000 Hz, we down‐sampled the data to 500 Hz. Additionally, we did not investigate the data where no low‐pass filter was applied. All other preprocessing steps were identical to study 1.

#### 4.1.1 Latency Extraction Techniques

Again, we compared all versions of a template matching algorithm resulting from the combinations of similarity measures, weighting windows, weighting functions, and penalty methods to traditional approaches such as peak latency, fractional area latency, and two variants of the fractional area latency algorithm proposed by Liesefeld (2018). For peak latency and 50% area latency algorithms, we used the respective measurement windows used by Sadus et al. (2024). See Figure 4 for an overview.

#### 4.1.2 Simulation Protocol

We chose to deviate from the simulation protocol used by Kiesel et al. (2008) in two ways.^2^ Firstly, we refrain from shifting the entire signal by a set distance and rather applied a linear transformation along the time‐dimension to the entire signal. Scaling the signal has the benefit that no missing values around the origin are created and that this approach may be more realistic as latency shifts are usually not constant for all components but rather increase in magnitude the later the component occurs (Luck 2014). Therefore, we chose to stretch the signal by a value λ using the same spline interpolation technique that we employ in the template matching process.

We introduced individual differences in the magnitude of the latency shift by randomly drawing the magnitude of the shift from a normal distribution with a mean of $\mu_{\lambda }=0.9$ and a standard deviation of $\sigma_{\lambda }=0.05$. We then set this random value as that subject's true latency shift parameter $\lambda_{j}$ for all iterations of the simulation and for all latency extraction methods.

At the beginning of each iteration, we randomly divided each subject's trials into control and experimental trials and then averaged them to generate control and experimental ERPs. We then stretched the experimental ERPs by that subject's experimental shift parameter $\lambda_{j}$. We applied all extraction algorithms to the control and experimental ERPs and recorded the recovered component latencies and fit statistics. Finally, we compute the recovered experimental shift of iteration i for person j $\lambda_{i,j}$ by dividing the latency in the control condition $l_{i,j|\text{control}}$ by the latency in the experimental condition $l_{i,j|\exp}$,

$$\lambda_{i,j}=\frac{l_{i,j|\text{control}}}{l_{i,j|\exp}}.$$

We repeated this process 100 times for each pre‐processing step, each time randomly splitting the available trials into a set of control and experimental trials. We removed latency estimates with bad template matching fit statistics (r<0.3) and unreasonable estimates of b (b≤0.5 or b≥1.9), subsequently, we removed latency estimates deviating more than three standard deviations from the mean across subjects. Then we averaged $\lambda_{i,j}$ over all simulations to obtain the recovered shift parameter per person $\hat{\lambda_{j}}$. This reduces the error variance that is introduced by the random split into control and experimental trials. Finally, we computed the intra‐class correlation focusing on absolute agreement between the true shift parameter $\lambda_{j}$ and the average recovered shift parameter $\hat{\lambda_{j}}$. To estimate the uncertainty due to sampling variability, we computed bootstrapped 95% confidence intervals for the mean algorithm performance. Specifically, we resampled participants with replacement 5000 times and recomputed all statistics for each resample. The confidence intervals correspond to the 2.5th and 97.5th percentiles of the resulting bootstrap distribution of mean performance.

### 4.2 Results

See Figure 8.

**FIGURE 8:**
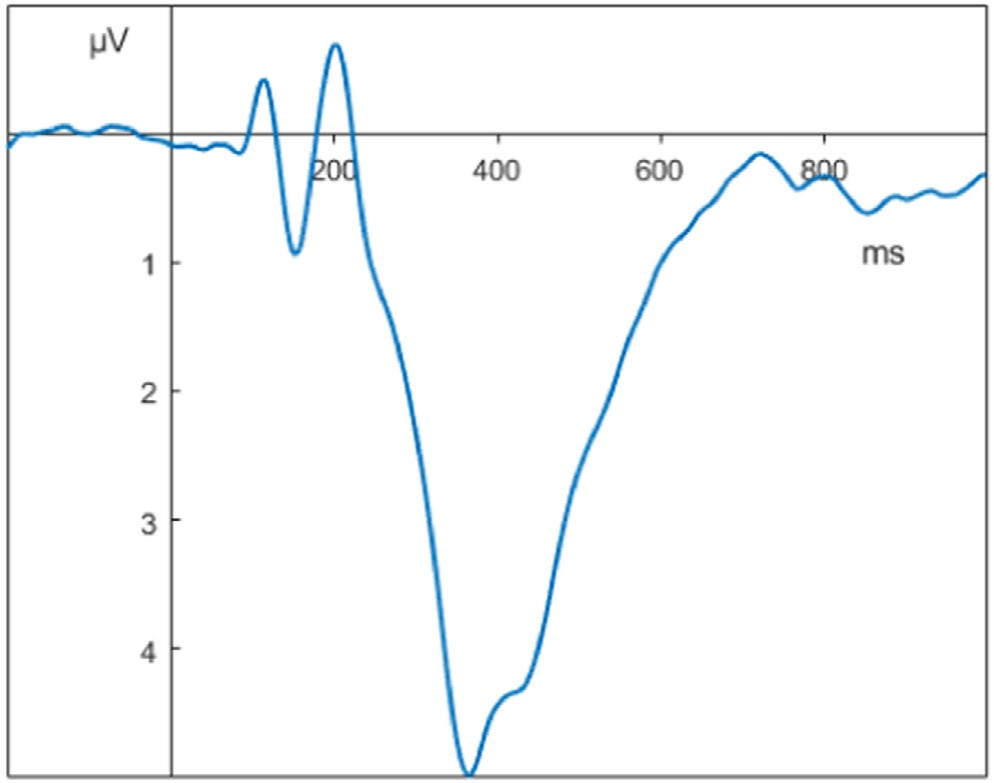
Grand averages of the Flanker task at Pz with a 32 Hz low‐pass filter.

#### 4.2.1 Descriptive Statistics

We removed subjects where less than 50% of the simulations yielded a valid latency estimate. An overview of the distribution of the fit statistic can be found in Figure A2.

The percentage of missing values per extraction method is reported in Table 4. Across preprocessing steps, weighting windows, weighting functions, and without a penalty, the MINSQ algorithm resulted in 15.26% missing values and the MAXCOR algorithm in 9.52% missing values. Employing normalized weights reduced the percentage of missing values to 8.82% and 4.89% for the MINSQ and MAXCOR algorithm, respectively. Penalizing more extreme transformation parameters reduced the percentage of missing values to 4.85% for the MINSQ algorithm and 3.57% for the MAXCOR algorithm across preprocessing steps, weighting windows, and weighting functions. The peak latency algorithm resulted in 2.56% missing values, 1.97% for the fractional area latency algorithm, and 2.55% and 1.64% missing values for the modified area latency algorithms using either the maximum peak amplitude (Liesefeld A) or constraining the measurement window by the on‐ and offset of the component (Liesefeld B), respectively.

**TABLE 4:** Missing values for different algorithms: simulation.

Approach	Weight	[200 700]		[250 700]		[250 900]		[300 600]	
		Penalized	None	Penalized	None	Penalized	None	Penalized	None
MAXCOR	None	4.88	8.16	4.92	8.19	4.91	8.19	4.83	8.15
	Hamming	2.33	8.52	2.96	11.68	4.99	10.82	4.00	19.46
	Tukey	2.75	8.37	3.53	11.79	6.19	11.35	4.14	18.07
	Normalized	1.81	5.16	1.61	4.94	1.47	5.18	1.85	4.29
MINSQ	None	4.56	18.75	4.54	18.91	4.56	18.77	4.58	18.88
	Hamming	3.43	12.28	4.57	14.52	9.63	24.07	4.65	13.31
	Tukey	4.54	14.43	5.84	16.71	9.75	23.25	6.18	15.04
	Normalized	2.82	9.51	2.66	8.91	2.63	8.87	2.67	8.00
Peak	None		1.04		1.06		2.56		1.76
Area	None		2.04		1.97		2.43		2.38
Liesefeld A	None		2.53		2.55		3.61		2.18
Liesefeld B	None		1.55		1.64		1.92		1.51

*Note:* Percent of missing values per algorithm. The rows indicate combinations of similarity measure and weighting function. The columns denote the measurement window and indicate if a penalty was used. MAXCOR and MINSQ refer to the template matching algorithms maximizing the correlation or minimizing the squared distance, respectively. Peak refers to the peak latency approach, area to a standard 50% fractional area latency approach. Liesefeld A and Liesefeld B refer to modified fractional area latency approaches proposed by Liesefeld et al. (2016, 2018). Liesefeld A uses 50% of the peak amplitude as the new baseline. Liesefeld B uses 30% of the peak amplitude as the baseline and additionally constrains the measurement window by the on‐ and offset of the component.

#### 4.2.2 Recovery

**FIGURE 9:**
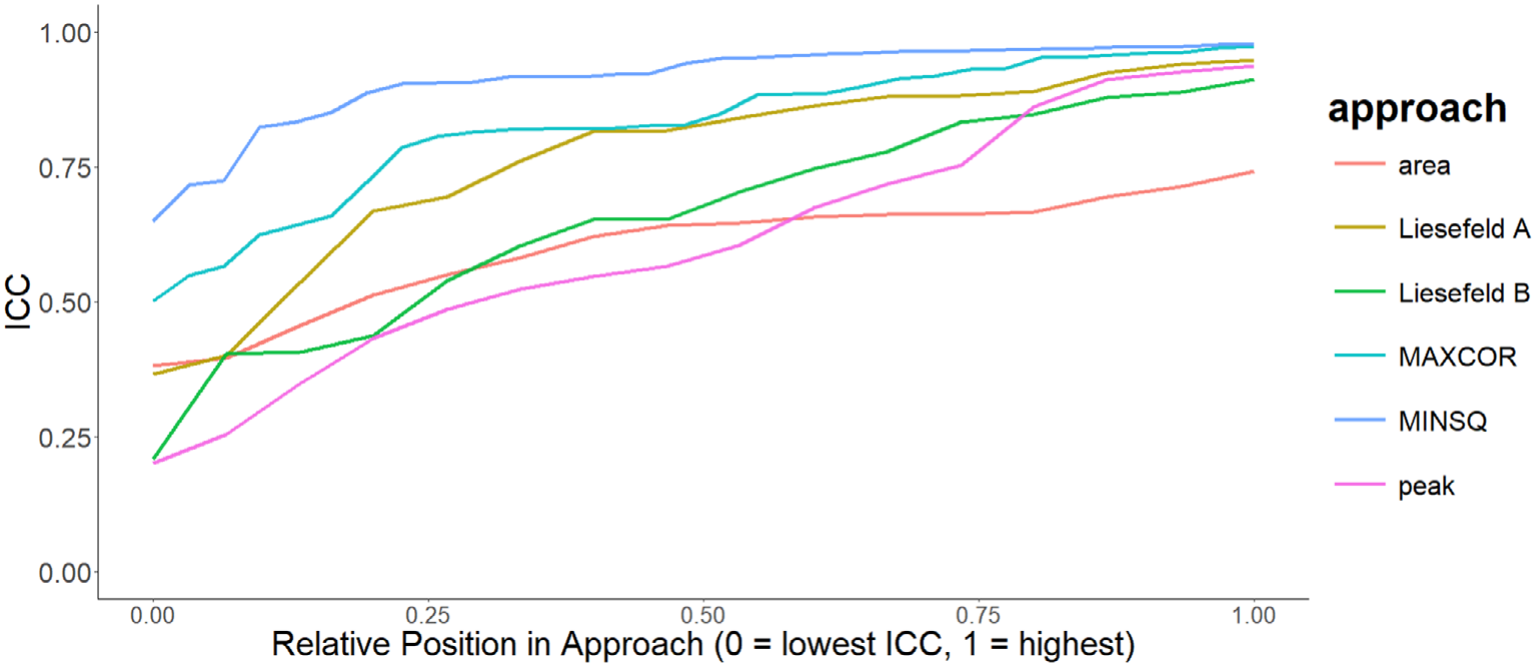
ICC estimates by extraction method. Overview of ICC focusing on absolute agreement between simulated shift and recovered shift by latency extraction method. The plot shows all ICC estimates across tasks, preprocessing settings and weighting windows ordered by size within a given approach. For the template matching approaches, only the normalized weighting function is displayed. MAXCOR and MINSQ refer to the template matching algorithms maximizing the correlation or minimizing the squared distance, respectively; peak refers to the peak latency approach, area to a standard 50% fractional area latency approach; Liesefeld A and Liesefeld B refer to modified fractional area latency approaches proposed by Liesefeld et al. (2016, 2018); Liesefeld A uses 50% of the peak amplitude as the new baseline; Liesefeld B uses 30% of the peak amplitude as the baseline and additionally constrains the measurement window by the on‐ and offset of the component.

Intra‐class correlations focusing on absolute agreement between true experimental shifts and average recovered shifts can be found in Table 5 and the full distribution is shown in Figure 9. The choice of weighting function had considerable impact on the recovery of simulated shifts. Hamming and Tukey weighting functions showed a mean $\mathrm{ICC}=0.57,95\%\mathrm{CI}\;\left[0.47,0.72\right]$ across preprocessing steps, weighting windows, and template matching algorithms. Using the grand average normalized weighting functions improved the mean $\mathrm{ICC}=0.87,95\%\mathrm{CI}\;\left[0.80,0.94\right]$.

**TABLE 5:** ICC between true and recovered experimental shift for different algorithms: simulation.

Approach	Weight	[200 700]		[250 700]		[250 900]		[300 600]	
		Penalized	None	Penalized	None	Penalized	None	Penalized	None
MAXCOR	None	0.94 [0.91, 0.96]	0.93 [0.87, 0.96]	0.94 [0.91, 0.96]	0.95 [0.92, 0.97]	0.93 [0.89, 0.95]	0.95 [0.92, 0.97]	0.93 [0.90, 0.96]	0.95 [0.92, 0.97]
	Hamming	0.54 [0.37, 0.80]	0.34 [0.06, 0.76]	0.58 [0.42, 0.81]	0.46 [0.15, 0.83]	0.78 [0.68, 0.86]	0.77 [0.65, 0.88]	0.22 [0.05, 0.50]	0.14 [−0.03, 0.46]
	Tukey	0.64 [0.48, 0.82]	0.33 [0.05, 0.77]	0.61 [0.46, 0.82]	0.33 [0.03, 0.80]	0.80 [0.70, 0.90]	0.81 [0.70, 0.91]	0.25 [0.10, 0.47]	0.15 [−0.01, 0.42]
	Normalized	0.82 [0.72, 0.96]	0.81 [0.62, 0.96]	0.86 [0.76, 0.96]	0.86 [0.74, 0.97]	0.82 [0.69, 0.97]	0.80 [0.66, 0.97]	0.86 [0.76, 0.94]	0.78 [0.66, 0.90]
MINSQ	None	0.86 [0.80, 0.91]	0.88 [0.79, 0.94]	0.86 [0.79, 0.91]	0.89 [0.81, 0.95]	0.84 [0.76, 0.90]	0.87 [0.78, 0.93]	0.84 [0.77, 0.90]	0.89 [0.80, 0.94]
	Hamming	0.74 [0.65, 0.82]	0.61 [0.45, 0.78]	0.67 [0.56, 0.78]	0.62 [0.50, 0.77]	0.75 [0.64, 0.84]	0.78 [0.69, 0.86]	0.55 [0.44, 0.67]	0.45 [0.32, 0.62]
	Tukey	0.79 [0.71, 0.87]	0.68 [0.53, 0.82]	0.69 [0.56, 0.83]	0.67 [0.55, 0.83]	0.77 [0.67, 0.86]	0.79 [0.69, 0.90]	0.56 [0.44, 0.70]	0.47 [0.33, 0.64]
	Normalized	0.93 [0.86, 0.97]	0.95 [0.91, 0.98]	0.93 [0.87, 0.98]	0.95 [0.91, 0.97]	0.91 [0.82, 0.98]	0.89 [0.79, 0.97]	0.84 [0.69, 0.94]	0.91 [0.87, 0.95]
Peak	None		0.64 [0.45, 0.91]		0.61 [0.41, 0.87]		0.80 [0.70, 0.91]		0.38 [0.19, 0.74]
Area	None		0.68 [0.51, 0.84]		0.63 [0.45, 0.80]		0.65 [0.53, 0.76]		0.44 [0.29, 0.58]
Liesefeld A	None		0.87 [0.74, 0.96]		0.85 [0.69, 0.95]		0.83 [0.72, 0.94]		0.50 [0.24, 0.80]
Liesefeld B	None		0.66 [0.53, 0.83]		0.66 [0.50, 0.85]		0.72 [0.57, 0.87]		0.58 [0.46, 0.84]

*Note:* Intra‐class correlations focusing on absolute agreement. Values in brackets provide bootstrapped 95% confidence intervals. The rows indicate combinations of similarity measure and weighting function. The columns denote the measurement window and indicate if a penalty was used. MAXCOR and MINSQ refer to the template matching algorithms maximizing the correlation or minimizing the squared distance, respectively. Peak refers to the peak latency approach, area to a standard 50% fractional area latency approach. Liesefeld A and Liesefeld B refer to modified fractional area latency approaches proposed by Liesefeld et al. (2016, 2018). Liesefeld A uses 50% of the peak amplitude as the new baseline. Liesefeld B uses 30% of the peak amplitude as the baseline and additionally constrains the measurement window by the on‐ and offset of the component.

Using the MINSQ or MAXCOR algorithm in conjunction with normalized weights yielded mean ICCs of $\mathrm{ICC}=0.91,95\%\mathrm{CI}\;\left[0.86,0.96\right]$ and $\mathrm{ICC}=0.83,95\%\mathrm{CI}\;\left[0.72,0.93\right]$, respectively. The peak latency algorithm showed a mean $\mathrm{ICC}=0.80,95\%\mathrm{CI}\;\left[0.70,0.91\right]$, the 50% area latency algorithm showed a mean $\mathrm{ICC}=0.63,95\%\mathrm{CI}\;\left[0.45,0.80\right]$, the modified area latency algorithm using the maximum amplitude (Liesefeld A) showed a mean $\mathrm{ICC}=0.85,95\%\mathrm{CI}\left[0.69,0.95\right],$ and the modified area latency algorithm constraining the measurement window by the on‐ and offset of the component (Liesefeld B) showed a mean $\mathrm{ICC}=0.66,95\%\mathrm{CI}\left[0.50,0.85\right]$.

### 4.3 Discussion

Similar to the empirical evaluation, using a normalized weighting function resulted in the best recovery of simulated latency shifts. Interestingly, both the MINSQ and MAXCOR approach performed well even when no weighting function was applied. These results underscore that Hamming and Tukey weighting functions are not optimal in the case of P3 latency extraction.

Importantly, only the MINSQ approach combined with a normalized weighting function consistently showed higher ICCs than other extraction algorithms (see Figure 9). While the MAXCOR approach with no weighting function showed good performance in the simulation, it showed worse performance in the empirical study. Only MINSQ and a normalized weighting function performed well in both the empirical evaluation as well as in the simulation. The performance benefits of the MINSQ approach seem to be especially pronounced in the worst 20% of ICCs across the multiverse pipelines. While this may change when applying the algorithm to other, in particular earlier ERP components, the results of the simulation study suggest using the MINSQ in combination with a normalized weighting function when aiming to extract P3 latencies.

## 5 General Discussion

Manual extraction of ERP latencies has so far proven more reliable and valid than algorithmic approaches (Sadus et al. 2024) but presents a time‐ and resource‐intensive process. The newly proposed template matching algorithm replicated human behavior and recovered simulated latency shifts better than any of the previous approaches like peak latency or fractional area latency. Template matching algorithms using normalized weights showed a mean ICC with manually extracted P3 latencies of $\mathrm{ICC}=0.89,95\%\mathrm{CI}\;\left[0.84,\;0.91\right]$ across tasks, filter settings, similarity measures, weighting windows, and penalties. This indicates that template matching algorithms are able to replicate manual extraction accurately while presenting a more objective and efficient approach to latency extraction. In addition, the MINSQ algorithm was able to closely recover simulated latency shifts. This algorithm using a normalized weighting function was best at recovering true latency shifts ($\mathrm{ICC}=0.91,95\%\mathrm{CI}\;\left[0.86,0.96\right]$) across filter settings, weighting windows, and penalties. Application of the algorithms based on the grand average can increase replicability and scalability. Furthermore, it significantly reduces the time and resources spent on latency extraction while proving consistently reliable and valid.

Notably, one of the modified fractional area latency algorithms (Liesefeld A) proposed by Liesefeld et al. (2016, 2018) performed slightly better than the template matching algorithms in the empirical study. While overlapping confidence intervals prohibit any strong conclusions about which algorithm is the absolute best, it is impressive that this algorithm matches the performance of template matching approaches with much lower complexity. The best template matching algorithms require the addition of a weighting function, whereas Liesefeld A is computationally simple and shows high agreement with a manual researcher. The fairest comparison, a template matching algorithm with minimal complexity—no weighting or penalty functions—performs much worse than Liesefeld A. Notably, some configurations of the fractional area latency algorithms proposed by Liesefeld et al. (2016, 2018) also performed poorly. We maintain that it is most informative to compare the best configurations of each approach head‐to‐head: the best template matching configuration vs. the best fractional area latency modification. In this case, well‐configured template matching approaches performed better in the simulation study and were more robust to researcher decisions such as the choice of measurement window. We believe that these performance benefits, coupled with the ability for efficient targeted manual intervention, illustrate the potential of the template matching approach.

The MINSQ approach in combination with normalized weights and a penalty for extreme values worked well across tasks, filter settings, and weighting windows. Nonetheless, the exact specification of similarity measures, weighting windows, weighting functions, and penalties depends on a variety of factors.

### 5.1 MAXCOR vs. MINSQ

In the empirical study, both the template matching algorithm maximizing the correlation (MAXCOR) and the algorithm minimizing the squared distance between template and signal (MINSQ) displayed high reliability and high agreement with a manual researcher when combined with a normalized weighting function. However, in the simulation MINSQ substantially outperformed MAXCOR in the recovery of simulated latency shifts. Additionally, in other applications where the parameter a, which scales the template along the amplitude‐axis, might be used to measure the amplitude of a component, the MAXCOR algorithm would be useless, as the correlation is invariant to linear transformations of the template's amplitude.

Focusing only on the empirical study, the MAXCOR approach slightly outperformed the MINSQ approach in replicating an expert ERP researcher. Importantly, even expert ERP researchers are not perfect when extracting ERP latencies, and the bootstrapped confidence intervals overlap substantially. Therefore, we place more emphasis on the results of the simulation, where the MINSQ approach yielded better results. In practice, both approaches performed well, and applying them will lead to improved quality in the extracted latencies. However, the results from the simulation study strongly suggest that the MINSQ approach is the preferred option. Therefore, we recommend using the MINSQ algorithm in future work and only retaining the correlation as a fit index.

### 5.2 Weighting Functions

We tested four different weighting functions. Not applying any weighting function did perform well in the simulation but performed poorly in empirical data. The Hamming and Tukey weighting functions performed poorly in both the empirical data and the simulation. Using the maximum‐normalized amplitude in the template to generate weights lead to the best results across the board. While the exact shape of the weighting function is open to discussion, these results support the notion that a weighting function that is based on the amplitude in the template itself will lead to the best results. We argue that these normalized weights are also the closest formalization of the process a human researcher employs when identifying ERP component latencies. The shape around the peak of the component is most important and the shape around the onset and offset of the components are less important. If there is a pronounced neighboring component, like the N2 in our case, researchers may use this as an additional indicator. The normalized weighting function best captures this behavior. Both the weighting function and weighting window can be freely specified by the user, allowing future researchers to implement custom weighting functions.

### 5.3 Weighting Windows

We investigated four different weighting windows in order to gauge the impact this has on our template matching algorithms. For both optimization methods and across tasks and filter settings, the weighting window did not impact the validity greatly. In some cases, the wide window (250–900 ms) seems to lead to slightly worse validity than the other windows. Similarly, the tight weighting window (300–600 ms) lead to worse indicators of validity in both the simulation and the empirical data. We therefore recommend specifying a weighting window in a similar manner to a measurement window for an area‐based latency algorithm. Any window that includes a part of the onset, the peak and a part of the offset should perform reasonably well.

This invariance of results to the weighting window is an additional benefit of template matching approaches. In contrast to traditional algorithms, the weighting window is not applied to each subject‐level ERP. Measurement windows, as used in traditional algorithms, may not fully include the component of interest in a given subject‐level ERP. Often, it is then the expert's job to readjust this measurement window for each individual. In the template matching algorithms, the weighting window is only applied to the template and then used to generate the weighing vector. Therefore, it will always include exactly those parts of the component that were previously specified by the researcher.

### 5.4 Optimization Bounds

The parameter bounds for *a* and *b* were selected based on practical considerations and empirical testing, rather than theoretical constraints. Specifically, these bounds were designed to prevent convergence on unrealistic values and to ensure stable performance of the algorithm across typical ERP datasets. While these bounds were tuned for our current application, they are likely suitable for most ERP studies, as individual latencies rarely diverge from the grand average by more than a factor of two. Importantly, the suitability of these bounds is determined not by the morphology of the ERP template itself, but by the expected variability in latency within the population or experimental context. Unless markedly greater inter‐individual or trial‐to‐trial latency variability is anticipated, we do not expect these bounds to require adjustment in other settings.

### 5.5 Penalty

Inclusion of a penalty term for extreme values of b during the optimization reduced the percentage of missing values greatly. We suspect that this is because extreme values are often the result of spurious high matches with very late or very early parts of the signal. This can occur in cases where the signal to noise ratio of a subject's ERP is low. Forcing the algorithm to converge on a more typical value thus leads to more reasonable latency estimates that also still generate acceptable fit statistics. Importantly, this is a penalty and not an upper/lower bound. The algorithm can converge on extreme values in cases where the latency of the component in the subject does deviate from the template greatly and less extreme values of b do not lead to significantly better similarity measures. Additionally, no penalty is being applied as long as $0.\bar{6}\leq b\leq 1.5$. Accordingly, no bias towards the mean is present during the optimization process for most ERPs.

A bias towards the mean when using the penalty would manifest in reduced validity. We did observe reduced validity in the simulation, but only for the MINSQ approach. In the empirical evaluation, the MINSQ approach matched the expert more closely when a penalty was used. The MAXCOR approach had higher validity in both the simulation and the empirical evaluation when the penalty was applied. Overall, we see no signs of a reduction in validity that a bias towards the mean would entail, which would have been evident across both optimization methods.

In practice, whether to apply a penalty should be determined by the degree of automation needed and the number of missing values that are tolerable. If there is no time to review cases with extreme values of b and missing values have to be avoided, a penalty term should be applied during optimization. This ensures a lower number of missing values without reducing validity. In order to ensure optimal validity, we suggest running the algorithm without a penalty term and then manually reviewing those cases with the worst fit statistics and the most extreme values of b.

Importantly, the penalty function outlined here represents a pragmatic approach that worked reasonably well in practice. While we find evidence supporting the general inclusion of a penalty function in the template matching process, its exact formulation is up for debate. The current implementation of the template matching algorithm allows researchers to specify their own penalty function. We believe that future research may seek more optimal penalty functions, but believe that a function penalizing extreme latency differences should improve the process in principle.

### 5.6 Fit Statistic

Template matching algorithms allow researchers to selectively review those subject‐level ERPs where the correlation between template and signal indicates low similarity. For example, researchers can use the algorithm to efficiently and objectively extract the majority of ERP latencies and only spend time on those cases with the worst fit statistics. Here, a human may be able to quantify some ERPs that the algorithm fails on, thus increasing the amount of usable data. By adjusting the cutoff after which manual review is necessary, researchers can be as liberal or conservative as they choose. The algorithm performs well without a review, but the validity of the extracted data will probably increase after human inspection.

We chose to use the correlation coefficient (MAXCOR) as the fit criterion for both methods, for several reasons. First, to ensure a consistent basis for comparison between approaches, it was necessary to apply the same thresholding metric across methods. Second, since our primary objective was latency estimation, we prioritized morphological similarity over amplitude similarity, which the correlation measure captures more effectively. Amplitude similarity, while potentially informative in other contexts, is less relevant for identifying valid latency estimates. Third, the correlation coefficient can be more easily interpreted than the distance metric and is independent of overall amplitude effects between experiments. Lastly, our empirical experience showed that using the MINSQ fit statistic often led to the erroneous rejection of valid ERPs, particularly those with correct waveform morphology but reduced or inverted amplitude. Because MINSQ is highly sensitive to amplitude differences, it may yield poor fit values even when the ERP shape closely matches the template. In contrast, low correlation values more reliably reflect genuine mismatches in waveform morphology, which was the key feature we aimed to filter. Our intent in applying a fit cut‐off was not to finely grade fit quality, but rather to exclude ERPs showing little or no morphological resemblance to the template. For this purpose, we found a conservative threshold (*r* < 0.3) to be both appropriate and effective. That said, we acknowledge that in future applications—particularly those involving manual review or where amplitude similarity is of interest—it may be beneficial to consider a combined fit criterion that incorporates both correlation and amplitude‐sensitive measures.

No other algorithms allow this type of efficient manual intervention or adjustable automatic rejection of the worst cases. In traditional approaches, subject‐level metrics of data quality, like the standardized measurement error (SME; Luck et al. 2021) may be used to exclude those subjects with insufficient quality metrics (Wascher et al. 2022). This does lead to improved results and may be considered as an alternative. However, there are several distinctions between SME and the fit statistic presented here. The SME is directly influenced by the number of trials (Zhang and Luck 2023) and the number of data points (Rodrigues et al. 2024), while the fit statistic generated by template matching approaches is only indirectly affected by the number of trials and unaffected by the number of data points. While the SME optimizes for no variance (Rodrigues et al. 2024), the present fit statistic optimizes for maximum covariance between the best estimate of the true signal and the present ERP. Finally, the correlation between template and signal provides a more intuitive quantification of data quality. It directly reflects the extent to which the component of interest is present in the data and how much it is corrupted by noise on a scale easily interpretable by researchers.

Importantly, noise in the sense of correlation between template and signal is any unsystematic difference in the covariance term between the signal and the transformed template. Trial‐to‐trial variability, which may be an important part of the neurocognitive processes underlying the task, will not influence the fit statistic as long as this variability occurs in most subjects. While the SME would classify this as noise, the fit statistic of the template matching algorithm allows for inter‐trial variability. For example, variability in the true latency of the component will lead to broader components in both the subject‐level ERPs as well as the template.

Lastly, we would like to provide some recommendations regarding the use of the fit statistic for manual review. These recommendations are only based on our practical experience and are limited to the current sample of tasks and subjects. In this paper, we used 0.3 as an arbitrary cutoff criterion. Based on our experience with the template matching algorithm, ERPs showing lower fit statistics seldom showed quantifiable ERPs. Because manual intervention was not possible due to the large amount of data, we chose this criterion as a conservative cutoff controlling our false alarm rate. In principle, we recommend a similar approach in new data. First, gather some experience with the present data and accompanying fit statistics before choosing a cutoff. Then, choose a cutoff based on the available resources for manual supervision. In our case, we observed that ERPs showing fit statistics greater than 0.7 seldom required manual intervention, indicating that especially fit statistics between 0.3 and 0.7 benefit from researcher intervention. However, we recommend inspecting a few sample cases and the distribution of fit statistics in new data before adopting a cutoff criterion. Then researchers should adjust the cutoff based on how tolerant the subsequent methods are regarding missing values or on the availability of resources for manual intervention. In cases with very few available resources, such as a large‐scale simulation, we recommend a lower cutoff than in cases where manual intervention is possible.

### 5.7 Limitations

Most importantly, this work is limited in scope. As a first step, we used template matching algorithms to extract P3 latencies only. In order to improve generalizability, we included three different tasks and varied the low‐pass filter during preprocessing. While the different template matching approaches differed in validity between the tasks and filter settings, we did not observe severe issues in any of these 15 different conditions, allowing the conclusion that the template matching algorithm performed well in the present sample of participants and tasks. Importantly, the morphology of the P3 in this study is more complex than in traditional P3 paradigms (e.g., oddball‐paradigms). Good performance in the present sample of tasks should generalize well to “simpler” P3 morphologies.

Nonetheless, the P3 is not a uniform component—its timing, shape, and size can vary notably across tasks, conditions, and individuals. This variability poses a challenge for latency measurement but also highlights a key strength of the template matching algorithm: its adaptability to the morphology of a component in a given context, something other extraction techniques lack.

Several characteristics of the data influence how well any latency extraction method performs. These include the distinctness of the component's shape, whether this shape varies between conditions, how prominent the component is relative to surrounding activity, and the degree of overlap with neighboring components. Systematic latency shifts in nearby components, subject‐to‐subject variability, and differing noise levels can all affect the psychometric qualities of latency estimates. While the P3's dominance in the signal may support better extraction performance, smaller or earlier components may require a more nuanced weighting function to isolate effectively.

Consequently, this study represents an initial step in validating the template matching approach. Future work should assess how well it generalizes to components with less distinct shapes or lower signal prominence, where performance may differ more substantially.

Additionally, we evaluated the latency estimation methods within the confines of a single, established preprocessing pipeline and only varied the low‐pass filter. While this approach enabled consistency and comparability with prior work (Sadus et al. 2024), it necessarily limits our ability to assess how different preprocessing choices—such as baseline correction, ICA thresholding, or artifact rejection criteria—might influence latency estimates. For instance, baseline correction can shift waveform amplitudes, potentially impacting area‐based metrics. Similarly, conservative ICA thresholds may exclude components containing early or mixed sources (Rodrigues et al. 2021), which could influence the morphological features used in latency estimation. Investigating the robustness of these methods across diverse preprocessing strategies is an important avenue for future research but was beyond the scope of the current work.

In conclusion, the results presented in this paper are limited to the P3 in a small sample of tasks. The generalizability of our approach remains to be studied in different tasks, samples, and components. We are eager to provide the necessary tools to simplify the application of the template matching algorithm to facilitate this future research.

This limited generalizability is further underscored by our use of descriptive statistics. As the present sample of tasks does not constitute a true random or representative sample of possible tasks, we believe that testing for significance in the population is ill advised. Consequently, differences in psychometric properties presented here can only be interpreted in the context of the present sample and do not allow generalization to other components and tasks. Additionally, bootstrapping revealed significant sampling variability in our point estimates. The confidence intervals of the best performing algorithms overlap substantially, especially in the empirical study. Hence, this work does not represent conclusive proof that template matching algorithms categorically outperform traditional approaches.

Instead, this study represents an initial step in validating the template matching approach. The algorithm's adequate reliability, strong agreement with an established measure of P3 latencies, and accurate recovery of simulated latency shifts support its construct validity and place it within the nomological network of P3 latency measures (Cronbach and Meehl 1955). Nonetheless, validation is not yet complete. Future research should continue the validation process by examining the algorithm's performance across a broader range of tasks and employing additional methods to further investigate its construct validity.

Lastly, the application of a template matching algorithm is more complex and computationally demanding than the previous algorithms. We want to address the complexity issue in future work and simplify the application of template matching algorithms to make them more accessible. Quantification of a single ERP takes around 0.05–0.2 s depending on the hardware. For extremely large data and low computing power, using the fractional area latency algorithm with the modifications proposed by Liesefeld (2018), using 50% of the peak amplitude as the new baseline, may be the better choice as this algorithm performed slightly better than template matching in the empirical study and yielded acceptable results in the simulation. However, with access to a high‐performance computing cluster running 25 nodes in parallel, we were able to quantify > 5 million ERPs using the template matching algorithm in under 24 h.

### 5.8 Future Research

This work presents a proof‐of‐concept that template matching algorithms can adequately recover P3 latencies. We had to restrict our analyses to only a small subset of possible parameter settings in the template matching algorithm. In order to facilitate future research investigating the robustness of parameter choices or aiming to provide recommendations for parameter settings, we have implemented the template matching algorithm to provide high flexibility in the definition of parameters. Researchers can freely specify weighting windows, weighting functions, penalty functions, and parameter boundaries, using either the set of weighting functions provided here or defining entirely new functions.

To improve accessibility, we plan to develop a user‐friendly graphical interface for the template matching algorithm (see the Supporting Material for more information on this interface S2). This interface will enable researchers to review algorithmic matches interactively and, if needed, adjust component latency estimates. We believe that the promising performance observed in this study highlights the potential of the template matching approach, particularly when implemented in an intuitive and easily applicable format.

As our primary aim was the development of the template matching approach and the discovery of reasonable parameter settings, we did not explore parameter settings in traditional approaches as thoroughly as the template matching ssuettings. We aimed to include the most common algorithms and apply them using reasonable measurement windows and also included the modified algorithms proposed by Liesefeld (2018) to study an extension of traditional approaches. However, even in traditional approaches, multiple researcher degrees of freedom remain. The *latency.m* function by Liesefeld (2018) allows a multitude of parameter settings that were not exhaustively explored. Similarly, fractional area latency approaches may differ in window width, location, percent area, baseline, etc. Additionally, fractional area latency approaches also allow the detection of problematic fits, for example, by using the total component area as an indicator of problematic ERPs. While this was out of the scope of the current study, we encourage future research to investigate the impact of parameter settings and outlier detection in traditional approaches.

One promising direction for future work is to integrate elements from *Residual Iteration Decomposition* (RIDE; Ouyang et al. 2011) for handling latency variability in event‐related potentials (ERPs). RIDE decomposes single‐trial EEG signals into component clusters and corrects for latency jitter using a cross‐correlation‐based alignment procedure. This approach enables the estimation of single‐trial latencies, which is particularly useful in paradigms where latency variability across trials or participants is a major concern.

While our approach shares some conceptual overlap with RIDE—namely the use of template matching—it differs in several key respects. Our method is based on matching a template transformed by stretching and compressing it along the time‐ and amplitude‐axis as opposed to cross‐correlation employed in RIDE, which only allows linear shifts and does not incorporate a weighting function. As such, our method trades some of RIDE's granularity for greater interpretability, lower computational complexity, and easier implementation. These qualities make our method particularly suitable for large datasets and applied research contexts where efficiency and transparency are critical.

Nonetheless, our approach may benefit from future enhancements inspired by RIDE. For instance, applying a Woody‐type alignment to individual ERPs before template construction could help mitigate latency jitter in the grand average, potentially improving the quality of the template.

In summary, while RIDE offers a powerful framework for detailed single‐trial latency estimation, our approach aims for a more accessible and computationally tractable solution. The fit statistic aids a case‐by‐case review of the algorithm's decisions not present in RIDE. Future research may explore hybrid models that retain the transparency and efficiency of our method while incorporating advanced latency correction strategies similar to those used in RIDE.

In the present study, we evaluated the use of template matching in the case of P3 latency extraction. The template matching approach is easily extendable to other components, tasks, and parameter settings, and this extension is a necessary step in future research to evaluate the algorithms' performance in a diverse set of applications. Importantly, the template matching approach is not limited to classical ERP signals. As a helpful reviewer pointed out, it is also possible to apply the template matching algorithm to difference waves, for example, to measure the onset latency of an effect. Similarly, it is also possible to use a subject‐level ERP as the template and apply it to measure single‐trial latencies, possibly incorporating some of the lessons learned by RIDE. While we have focused on the latency extraction of subject‐level P3 latencies, the method itself can be applied to any signal where the researcher can specify both a template and a timepoint 0, from which the signal can be stretched and compressed.

## 6 Conclusion

Template matching algorithms using the grand average as a dynamic template were able to extract P3 latencies better than any previous algorithm in the combination of empirical data and simulation. The algorithm minimizing the distance between template and ERP (MINSQ) showed high agreement with latencies extracted by an expert researcher and the best recovery of true simulated latency shifts. The algorithm based on maximizing the correlation (MAXCOR) performed well in the empirical study but was inferior to the MINSQ approach in the simulation study. One of the modifications proposed by Liesefeld et al. (2016), using 50% of the peak amplitude as the baseline instead of zero, seemed to be the best available “traditional” alternative. It yielded comparable results to the best template matching algorithm in the empirical study but worse results than the best template matching algorithm in the simulation study. However, large confidence intervals due to sampling variability highlight the need for further studies to inspect performance differences.

The MINSQ approach in combination with normalized weights works well across tasks, filter settings, weighting windows, and penalties. The algorithm also provides a fit statistic that may be used to automatically discard those ERPs with low matches to the template. Importantly, these fit statistics may also be used to flag certain ERPs for later manual review, allowing for efficient resource allocation during manual intervention.

The exact specification of which similarity measure to use, which weighting function to apply, whether to apply a penalty, and which cutoff to use for the fit statistic is up to each individual researcher. We recommend using the MINSQ approach with normalized weights and a weighting window that includes the on‐ and offset of the component. If no manual inspection is possible, we recommend using a penalty for extreme values of b during the optimization process. Otherwise, we recommend using the unpenalized MINSQ approach and manually reviewing only cases with the worst fits and most extreme values of b. We have provided additional Supporting Materials that offer detailed explanations of parameter choices, algorithmic features (such as handling multiple peaks), and practical considerations for applying the template‐matching approach.

We showed that this template matching algorithm improves objectivity and efficiency while maintaining consistently good reliability and superior validity over previous approaches. While the performance benefits in the empirical evaluation are modest, we argue that even small gains—when paired with the efficiency and transparency of template matching—can yield meaningful improvements in research practice. Importantly, the accompanying fit statistic allows researchers to prioritize cases for manual review, a feature that is largely absent in other latency estimation methods.

This algorithm could be used as a final step for latency estimation in multiverse studies (Sadus et al. 2024; Zhang and Luck 2023) and other automated pipelines (Clayson et al. 2021; Rodrigues et al. 2021; Wascher et al. 2022). The complete set of functions necessary to run the algorithm and example data and scripts are available on GitHub (https://github.com/SLesche/template‐matching‐paper). We hope to demonstrate the efficacy of template matching algorithms for other components in future work.

## Author Contributions

**Anna‐Lena Schubert:** writing – review and editing, data curation, funding acquisition, resources, supervision. **Christoph Löffler:** software, data curation, investigation, project administration, writing – review and editing. **Dirk Hagemann:** conceptualization, resources, supervision, writing – review and editing. **Kathrin Sadus:** data curation, validation, writing – review and editing. **Sven Lesche:** conceptualization, data curation, formal analysis, methodology, project administration, software, validation, writing – review and editing, writing – original draft, visualization.

## Funding

This work was supported by the German Research Foundation (DFG) (grant number SCHU 3266/1‐1 and SCHU 3266/2‐1). Open Access funding enabled and organized byProjekt DEAL.

## Ethics Statement

The study protocol has been approved by the local ethics committee of the Faculty of Behavioral and Cultural Studies of the Ruprecht‐Karls‐University Heidelberg.

## Conflicts of Interest

The authors declare no conflicts of interest.

## Supporting information


**Appendix S1:** psyp70212‐sup‐0001‐Appendix.docx.

**Appendix S2:** psyp70212‐sup‐0002‐Supplemental Materials.pdf.

## Data Availability

Code to replicate all analysis is available on GitHub (https://github.com/SLesche/template‐matching‐paper). Raw data is available upon request to the first author of this paper.

## Endnotes

^1^
Additionally, we use the Optimization Toolbox, the Global Optimization Toolbox, and the Signal Processing Toolbox for the template matching algorithm. Furthermore, the simulation relied on the Database Toolbox, and the Parallel Computing Toolbox.

^2^
Following the helpful recommendation of our reviewers, we also employed a constant shift in order to rule out issues caused by the similarity between the recovery algorithm and the simulation approach. The results of this constant shift simulation can be found in the appendix in Table A8. Qualitatively, the results did not differ from those presented here, except that the MAXCOR approach performs as well as the MINSQ approach and is no longer inferior.
