# Supplementary material for: Exploring a Dynamic Template Matching Algorithm for the Automatic Extraction of P3 Latencies
Source: Psychophysiology. 2025 Dec 23;63(1):e70212. doi: 10.1111/psyp.70212 (PMC12728138; doi:10.1111/psyp.70212)
Supplement: Supplementary file 1 — Appendix S1: psyp70212‐sup‐0001‐Appendix.docx. [file PSYP-63-e70212-s001.docx]

# Appendix

# Empirical data

## Missing values

**Table A1.**:

*Missing values of different algorithms by task*

| approach | weight | task | [200 700] | | [250 700] | | [250 900] | | [300 600] | |
| --- | --- | --- | --- | --- | --- | --- | --- | --- | --- | --- |
|  |  |  | penalized | none | penalized | none | penalized | none | penalized | none |
| MAXCOR | none | flanker | 4.17 | 5.50 | 4.17 | 5.50 | 4.17 | 5.50 | 4.17 | 5.50 |
|  | none | nback | 7.17 | 12.33 | 7.17 | 12.33 | 7.17 | 12.33 | 7.17 | 12.33 |
|  | none | switching | 6.17 | 9.50 | 6.17 | 9.50 | 6.17 | 9.50 | 6.17 | 9.50 |
|  | Hamming | flanker | 1.66 | 8.33 | 2.83 | 14.17 | 4.83 | 8.33 | 5.33 | 21.67 |
|  | Hamming | nback | 6.17 | 13.67 | 5.00 | 12.17 | 7.00 | 9.16 | 5.50 | 15.83 |
|  | Hamming | switching | 3.17 | 27.50 | 1.50 | 26.33 | 0.83 | 10.17 | 4.50 | 40.33 |
|  | Tukey | flanker | 2.00 | 7.17 | 4.00 | 12.17 | 4.83 | 8.33 | 5.17 | 22.00 |
|  | Tukey | nback | 6.33 | 15.50 | 5.83 | 11.83 | 7.50 | 8.67 | 4.33 | 13.67 |
|  | Tukey | switching | 4.83 | 25.50 | 1.83 | 23.67 | 2.17 | 9.00 | 4.50 | 37.50 |
|  | normalized | flanker | 2.17 | 4.50 | 2.00 | 4.33 | 2.17 | 4.83 | 2.67 | 4.00 |
|  | normalized | nback | 4.33 | 12.33 | 4.33 | 12.00 | 3.83 | 12.33 | 4.67 | 11.00 |
|  | normalized | switching | 1.00 | 15.00 | 0.83 | 14.67 | 0.50 | 16.67 | 1.83 | 11.83 |
| MINSQ | none | flanker | 3.33 | 14.17 | 3.33 | 14.17 | 3.33 | 14.17 | 3.33 | 14.17 |
|  | none | nback | 7.33 | 22.00 | 7.33 | 22.00 | 7.33 | 22.00 | 7.33 | 22.00 |
|  | none | switching | 9.34 | 27.33 | 9.34 | 27.33 | 9.34 | 27.33 | 9.34 | 27.33 |
|  | Hamming | flanker | 2.50 | 8.16 | 4.00 | 8.83 | 7.83 | 19.33 | 3.50 | 8.17 |
|  | Hamming | nback | 9.83 | 22.67 | 8.17 | 24.67 | 12.33 | 32.17 | 11.17 | 24.83 |
|  | Hamming | switching | 4.83 | 27.83 | 2.33 | 25.00 | 8.50 | 28.67 | 9.83 | 30.67 |
|  | Tukey | flanker | 3.17 | 10.33 | 4.17 | 10.67 | 7.83 | 19.00 | 3.17 | 8.83 |
|  | Tukey | nback | 11.17 | 23.50 | 9.50 | 24.83 | 10.83 | 27.67 | 9.67 | 23.83 |
|  | Tukey | switching | 10.00 | 31.83 | 7.00 | 29.00 | 10.33 | 30.50 | 9.67 | 30.83 |
|  | normalized | flanker | 2.00 | 7.33 | 2.00 | 7.33 | 2.00 | 7.83 | 2.83 | 5.67 |
|  | normalized | nback | 6.00 | 18.83 | 6.83 | 18.83 | 7.17 | 18.67 | 6.50 | 17.67 |
|  | normalized | switching | 2.50 | 21.67 | 2.00 | 21.17 | 1.67 | 25.33 | 3.33 | 19.67 |
| peak | none | flanker |  | 1.83 |  | 1.83 |  | 2.33 |  | 0.67 |
|  | none | nback |  | 1.83 |  | 1.83 |  | 2.16 |  | 2.00 |
|  | none | switching |  | 0.00 |  | 0.00 |  | 0.83 |  | 1.17 |
| area | none | flanker |  | 2.67 |  | 2.67 |  | 3.50 |  | 1.83 |
|  | none | nback |  | 2.00 |  | 2.00 |  | 2.66 |  | 1.50 |
|  | none | switching |  | 3.17 |  | 3.50 |  | 4.17 |  | 3.83 |
| Liesefeld A | none | flanker |  | 3.50 |  | 3.50 |  | 4.00 |  | 2.50 |
|  | none | nback |  | 3.50 |  | 3.50 |  | 3.50 |  | 1.50 |
|  | none | switching |  | 3.50 |  | 3.83 |  | 4.00 |  | 4.33 |
| Liesefeld B | none | flanker |  | 1.66 |  | 1.66 |  | 2.33 |  | 1.50 |
|  | none | nback |  | 1.00 |  | 1.00 |  | 1.83 |  | 0.50 |
|  | none | switching |  | 0.00 |  | 0.00 |  | 0.00 |  | 0.00 |
| *Note.* Percent of missing values per algorithm. The rows indicate combinations of similarity measures and weighting functions. The columns denote the measurement window and indicate if a penalty was used. MAXCOR and MINSQ refer to the template matching algorithms maximizing the correlation or minimizing the squared distance, respectively. peak refers to the peak latency approach, area to a standard 50% fractional area latency approach. Liesefeld A and Liesefeld B refer to modified fractional area latency approaches proposed by Liesefeld (2016; 2018). Liesefeld A uses 50% of the peak amplitude as the new baseline. Liesefeld B uses 30% of the peak amplitude as the baseline and additionally constrains the measurement window by the on- and offset of the component. | | | | | | | | | | |

**Table A2.**:

*Missing values of different algorithms by filter setting*

| approach | weight | filter | [200 700] | | [250 700] | | [250 900] | | [300 600] | |
| --- | --- | --- | --- | --- | --- | --- | --- | --- | --- | --- |
|  |  |  | penalized | none | penalized | none | penalized | none | penalized | none |
| MAXCOR | none | 0 | 5.83 | 8.61 | 5.83 | 8.61 | 5.83 | 8.61 | 5.83 | 8.61 |
|  | none | 4 | 6.11 | 9.17 | 6.11 | 9.17 | 6.11 | 9.17 | 6.11 | 9.17 |
|  | none | 8 | 5.56 | 9.45 | 5.56 | 9.45 | 5.56 | 9.45 | 5.56 | 9.45 |
|  | none | 16 | 5.56 | 8.89 | 5.56 | 8.89 | 5.56 | 8.89 | 5.56 | 8.89 |
|  | none | 32 | 6.11 | 9.45 | 6.11 | 9.45 | 6.11 | 9.45 | 6.11 | 9.45 |
|  | Hamming | 0 | 3.61 | 16.39 | 2.50 | 18.05 | 3.61 | 9.17 | 3.61 | 26.11 |
|  | Hamming | 4 | 3.89 | 13.33 | 3.06 | 13.06 | 5.83 | 8.89 | 5.83 | 19.17 |
|  | Hamming | 8 | 4.17 | 17.50 | 3.33 | 19.45 | 4.17 | 9.72 | 5.00 | 28.33 |
|  | Hamming | 16 | 3.61 | 18.61 | 3.61 | 19.44 | 4.17 | 9.44 | 5.83 | 29.45 |
|  | Hamming | 32 | 3.05 | 16.67 | 3.06 | 17.78 | 3.33 | 8.89 | 5.28 | 26.67 |
|  | Tukey | 0 | 4.72 | 15.28 | 3.33 | 15.83 | 5.00 | 9.17 | 2.78 | 23.89 |
|  | Tukey | 4 | 5.28 | 12.78 | 3.89 | 12.78 | 5.56 | 8.89 | 6.39 | 17.50 |
|  | Tukey | 8 | 4.72 | 18.06 | 4.17 | 17.78 | 4.72 | 8.33 | 5.28 | 26.67 |
|  | Tukey | 16 | 3.89 | 17.22 | 4.16 | 17.22 | 4.44 | 8.61 | 5.28 | 27.78 |
|  | Tukey | 32 | 3.33 | 16.94 | 3.89 | 15.83 | 4.44 | 8.33 | 3.61 | 26.11 |
|  | normalized | 0 | 2.50 | 11.95 | 1.67 | 11.11 | 1.67 | 12.78 | 2.50 | 9.72 |
|  | normalized | 4 | 3.33 | 7.78 | 3.61 | 7.50 | 3.06 | 7.78 | 4.17 | 7.22 |
|  | normalized | 8 | 2.78 | 10.55 | 2.78 | 10.28 | 2.22 | 10.83 | 3.61 | 8.33 |
|  | normalized | 16 | 1.94 | 11.39 | 2.22 | 11.67 | 1.94 | 12.50 | 2.50 | 10.00 |
|  | normalized | 32 | 1.94 | 11.39 | 1.67 | 11.11 | 1.94 | 12.50 | 2.50 | 9.45 |
| MINSQ | none | 0 | 6.95 | 22.22 | 6.95 | 22.22 | 6.95 | 22.22 | 6.95 | 22.22 |
|  | none | 4 | 6.11 | 19.72 | 6.11 | 19.72 | 6.11 | 19.72 | 6.11 | 19.72 |
|  | none | 8 | 6.39 | 20.56 | 6.39 | 20.56 | 6.39 | 20.56 | 6.39 | 20.56 |
|  | none | 16 | 6.95 | 22.50 | 6.95 | 22.50 | 6.95 | 22.50 | 6.95 | 22.50 |
|  | none | 32 | 6.95 | 20.83 | 6.95 | 20.83 | 6.95 | 20.83 | 6.95 | 20.83 |
|  | Hamming | 0 | 5.28 | 16.94 | 4.72 | 17.78 | 10.28 | 26.39 | 10.83 | 19.45 |
|  | Hamming | 4 | 5.83 | 21.94 | 4.72 | 21.95 | 9.17 | 25.83 | 5.55 | 23.61 |
|  | Hamming | 8 | 5.83 | 21.39 | 5.28 | 21.11 | 9.72 | 27.22 | 6.67 | 21.67 |
|  | Hamming | 16 | 6.11 | 19.16 | 4.44 | 19.44 | 9.44 | 27.50 | 8.61 | 21.39 |
|  | Hamming | 32 | 5.55 | 18.33 | 5.00 | 17.22 | 9.17 | 26.67 | 9.17 | 20.00 |
|  | Tukey | 0 | 7.78 | 20.55 | 7.78 | 20.00 | 9.44 | 24.17 | 9.17 | 20.28 |
|  | Tukey | 4 | 8.05 | 22.50 | 6.39 | 22.50 | 10.83 | 27.22 | 7.22 | 25.56 |
|  | Tukey | 8 | 8.61 | 24.44 | 6.39 | 23.89 | 9.17 | 26.39 | 6.67 | 20.00 |
|  | Tukey | 16 | 7.78 | 21.95 | 6.95 | 21.39 | 10.00 | 25.83 | 7.22 | 20.83 |
|  | Tukey | 32 | 8.33 | 20.00 | 6.94 | 19.72 | 8.89 | 25.00 | 7.22 | 19.17 |
|  | normalized | 0 | 3.06 | 14.72 | 3.05 | 13.89 | 3.06 | 16.39 | 3.06 | 14.72 |
|  | normalized | 4 | 4.17 | 15.28 | 4.72 | 14.72 | 3.89 | 16.11 | 5.00 | 12.78 |
|  | normalized | 8 | 3.89 | 17.22 | 3.89 | 16.67 | 4.72 | 18.33 | 5.00 | 12.78 |
|  | normalized | 16 | 3.05 | 15.83 | 3.05 | 16.39 | 3.05 | 17.50 | 3.89 | 15.83 |
|  | normalized | 32 | 3.33 | 16.67 | 3.33 | 17.22 | 3.33 | 18.06 | 4.17 | 15.55 |
| peak | none | 0 |  | 1.11 |  | 1.11 |  | 1.66 |  | 0.56 |
|  | none | 4 |  | 1.11 |  | 1.11 |  | 1.67 |  | 3.06 |
|  | none | 8 |  | 1.67 |  | 1.67 |  | 1.94 |  | 1.11 |
|  | none | 16 |  | 1.11 |  | 1.11 |  | 1.66 |  | 0.83 |
|  | none | 32 |  | 1.11 |  | 1.11 |  | 1.94 |  | 0.83 |
| area | none | 0 |  | 2.22 |  | 2.22 |  | 3.06 |  | 1.67 |
|  | none | 4 |  | 3.89 |  | 4.16 |  | 4.72 |  | 4.16 |
|  | none | 8 |  | 2.50 |  | 2.78 |  | 3.61 |  | 2.78 |
|  | none | 16 |  | 2.22 |  | 2.22 |  | 2.78 |  | 1.67 |
|  | none | 32 |  | 2.22 |  | 2.22 |  | 3.06 |  | 1.67 |
| Liesefeld A | none | 0 |  | 2.78 |  | 2.78 |  | 3.06 |  | 1.67 |
|  | none | 4 |  | 5.00 |  | 5.28 |  | 5.83 |  | 4.72 |
|  | none | 8 |  | 3.61 |  | 3.89 |  | 4.17 |  | 3.05 |
|  | none | 16 |  | 3.33 |  | 3.33 |  | 3.33 |  | 2.50 |
|  | none | 32 |  | 2.78 |  | 2.78 |  | 2.78 |  | 1.94 |
| Liesefeld B | none | 0 |  | 0.83 |  | 0.83 |  | 1.66 |  | 0.83 |
|  | none | 4 |  | 0.83 |  | 0.83 |  | 0.83 |  | 0.28 |
|  | none | 8 |  | 1.11 |  | 1.11 |  | 1.39 |  | 0.56 |
|  | none | 16 |  | 0.83 |  | 0.83 |  | 1.39 |  | 0.83 |
|  | none | 32 |  | 0.83 |  | 0.83 |  | 1.66 |  | 0.83 |
| *Note.* Percent of missing values per algorithm. The rows indicate combinations of similarity measures and weighting function. The columns denote the measurement window and indicate if a penalty was used. MAXCOR and MINSQ refer to the template matching algorithms maximizing the correlation or minimizing the squared distance, respectively. peak refers to the peak latency approach, area to a standard 50% fractional area latency approach. Liesefeld A and Liesefeld B refer to modified fractional area latency approaches proposed by Liesefeld (2016; 2018). Liesefeld A uses 50% of the peak amplitude as the new baseline. Liesefeld B uses 30% of the peak amplitude as the baseline and additionally constrains the measurement window by the on- and offset of the component. | | | | | | | | | | |

## Reliability

**Table A3.**

*Reliability of different algorithms by task*

| approach | weight | task | [200 700] | | [250 700] | | [250 900] | | [300 600] | |
| --- | --- | --- | --- | --- | --- | --- | --- | --- | --- | --- |
|  |  |  | penalized | none | penalized | none | penalized | none | penalized | none |
| MAXCOR | none | flanker | 0.90 | 0.92 | 0.90 | 0.92 | 0.90 | 0.92 | 0.90 | 0.92 |
|  | none | nback | 0.87 | 0.90 | 0.87 | 0.90 | 0.87 | 0.90 | 0.87 | 0.90 |
|  | none | switching | 0.86 | 0.83 | 0.86 | 0.83 | 0.86 | 0.83 | 0.86 | 0.83 |
|  | Hamming | flanker | 0.94 | 0.93 | 0.94 | 0.93 | 0.93 | 0.92 | 0.90 | 0.89 |
|  | Hamming | nback | 0.87 | 0.89 | 0.76 | 0.76 | 0.87 | 0.85 | 0.78 | 0.80 |
|  | Hamming | switching | 0.87 | 0.91 | 0.83 | 0.81 | 0.77 | 0.74 | 0.74 | 0.66 |
|  | Tukey | flanker | 0.93 | 0.93 | 0.93 | 0.92 | 0.93 | 0.93 | 0.87 | 0.89 |
|  | Tukey | nback | 0.88 | 0.88 | 0.82 | 0.78 | 0.83 | 0.83 | 0.82 | 0.82 |
|  | Tukey | switching | 0.88 | 0.88 | 0.84 | 0.85 | 0.81 | 0.72 | 0.75 | 0.72 |
|  | normalized | flanker | 0.93 | 0.93 | 0.93 | 0.92 | 0.92 | 0.93 | 0.91 | 0.91 |
|  | normalized | nback | 0.82 | 0.82 | 0.82 | 0.83 | 0.83 | 0.84 | 0.83 | 0.80 |
|  | normalized | switching | 0.86 | 0.87 | 0.86 | 0.87 | 0.89 | 0.84 | 0.88 | 0.83 |
| MINSQ | none | flanker | 0.90 | 0.92 | 0.90 | 0.92 | 0.90 | 0.92 | 0.90 | 0.92 |
|  | none | nback | 0.87 | 0.91 | 0.87 | 0.91 | 0.87 | 0.91 | 0.87 | 0.91 |
|  | none | switching | 0.86 | 0.83 | 0.86 | 0.83 | 0.86 | 0.83 | 0.86 | 0.83 |
|  | Hamming | flanker | 0.94 | 0.94 | 0.90 | 0.88 | 0.88 | 0.87 | 0.93 | 0.89 |
|  | Hamming | nback | 0.86 | 0.80 | 0.91 | 0.84 | 0.88 | 0.85 | 0.82 | 0.82 |
|  | Hamming | switching | 0.86 | 0.84 | 0.87 | 0.81 | 0.80 | 0.68 | 0.84 | 0.81 |
|  | Tukey | flanker | 0.93 | 0.94 | 0.92 | 0.87 | 0.90 | 0.83 | 0.93 | 0.89 |
|  | Tukey | nback | 0.90 | 0.88 | 0.87 | 0.86 | 0.86 | 0.88 | 0.82 | 0.83 |
|  | Tukey | switching | 0.85 | 0.86 | 0.85 | 0.84 | 0.82 | 0.72 | 0.88 | 0.83 |
|  | normalized | flanker | 0.92 | 0.91 | 0.92 | 0.91 | 0.92 | 0.91 | 0.91 | 0.91 |
|  | normalized | nback | 0.85 | 0.86 | 0.85 | 0.85 | 0.86 | 0.87 | 0.86 | 0.87 |
|  | normalized | switching | 0.89 | 0.83 | 0.90 | 0.83 | 0.90 | 0.83 | 0.85 | 0.79 |
| peak | none | flanker |  | 0.81 |  | 0.85 |  | 0.84 |  | 0.80 |
|  | none | nback |  | 0.74 |  | 0.73 |  | 0.68 |  | 0.73 |
|  | none | switching |  | 0.85 |  | 0.86 |  | 0.81 |  | 0.78 |
| area | none | flanker |  | 0.93 |  | 0.93 |  | 0.90 |  | 0.92 |
|  | none | nback |  | 0.88 |  | 0.88 |  | 0.88 |  | 0.85 |
|  | none | switching |  | 0.89 |  | 0.88 |  | 0.81 |  | 0.90 |
| Liesefeld A | none | flanker |  | 0.91 |  | 0.93 |  | 0.89 |  | 0.92 |
|  | none | nback |  | 0.84 |  | 0.84 |  | 0.78 |  | 0.85 |
|  | none | switching |  | 0.91 |  | 0.90 |  | 0.89 |  | 0.89 |
| Liesefeld B | none | flanker |  | 0.80 |  | 0.84 |  | 0.89 |  | 0.85 |
|  | none | nback |  | 0.80 |  | 0.80 |  | 0.70 |  | 0.79 |
|  | none | switching |  | 0.89 |  | 0.87 |  | 0.87 |  | 0.84 |
| *Note.* Reliability has been estimated by two-part coefficient alpha. The rows indicate combinations of similarity measures and weighting functions. The columns denote the measurement window and indicate if a penalty was used. MAXCOR and MINSQ refer to the template matching algorithms maximizing the correlation or minimizing the squared distance, respectively. peak refers to the peak latency approach, area to a standard 50% fractional area latency approach. Liesefeld A and Liesefeld B refer to modified fractional area latency approaches proposed by Liesefeld (2016; 2018). Liesefeld A uses 50% of the peak amplitude as the new baseline. Liesefeld B uses 30% of the peak amplitude as the baseline and additionally constrains the measurement window by the on- and offset of the component. | | | | | | | | | | |

**Table A4**.

*Reliability of different algorithms by filter setting*

| approach | weight | filter | [200 700] | | [250 700] | | [250 900] | | [300 600] | |
| --- | --- | --- | --- | --- | --- | --- | --- | --- | --- | --- |
|  |  |  | penalized | none | penalized | none | penalized | none | penalized | none |
| MAXCOR | none | 0 | 0.88 | 0.87 | 0.88 | 0.87 | 0.88 | 0.87 | 0.88 | 0.87 |
|  | none | 4 | 0.89 | 0.88 | 0.89 | 0.88 | 0.89 | 0.88 | 0.89 | 0.88 |
|  | none | 8 | 0.89 | 0.90 | 0.89 | 0.90 | 0.89 | 0.90 | 0.89 | 0.90 |
|  | none | 16 | 0.86 | 0.89 | 0.86 | 0.89 | 0.86 | 0.89 | 0.86 | 0.89 |
|  | none | 32 | 0.88 | 0.88 | 0.88 | 0.88 | 0.88 | 0.88 | 0.88 | 0.88 |
|  | Hamming | 0 | 0.88 | 0.89 | 0.83 | 0.82 | 0.84 | 0.86 | 0.77 | 0.76 |
|  | Hamming | 4 | 0.91 | 0.89 | 0.85 | 0.85 | 0.88 | 0.85 | 0.87 | 0.86 |
|  | Hamming | 8 | 0.91 | 0.93 | 0.85 | 0.86 | 0.85 | 0.82 | 0.85 | 0.78 |
|  | Hamming | 16 | 0.90 | 0.91 | 0.84 | 0.80 | 0.86 | 0.81 | 0.79 | 0.80 |
|  | Hamming | 32 | 0.86 | 0.92 | 0.86 | 0.85 | 0.85 | 0.84 | 0.75 | 0.71 |
|  | Tukey | 0 | 0.88 | 0.87 | 0.84 | 0.83 | 0.86 | 0.84 | 0.79 | 0.79 |
|  | Tukey | 4 | 0.91 | 0.89 | 0.87 | 0.87 | 0.88 | 0.86 | 0.88 | 0.87 |
|  | Tukey | 8 | 0.91 | 0.92 | 0.86 | 0.87 | 0.83 | 0.80 | 0.81 | 0.80 |
|  | Tukey | 16 | 0.90 | 0.92 | 0.89 | 0.82 | 0.84 | 0.82 | 0.82 | 0.83 |
|  | Tukey | 32 | 0.89 | 0.89 | 0.85 | 0.87 | 0.86 | 0.81 | 0.76 | 0.76 |
|  | normalized | 0 | 0.84 | 0.83 | 0.84 | 0.83 | 0.82 | 0.86 | 0.85 | 0.85 |
|  | normalized | 4 | 0.91 | 0.91 | 0.91 | 0.90 | 0.90 | 0.91 | 0.90 | 0.91 |
|  | normalized | 8 | 0.89 | 0.89 | 0.90 | 0.89 | 0.91 | 0.87 | 0.90 | 0.84 |
|  | normalized | 16 | 0.89 | 0.89 | 0.86 | 0.88 | 0.89 | 0.86 | 0.87 | 0.84 |
|  | normalized | 32 | 0.83 | 0.86 | 0.82 | 0.88 | 0.88 | 0.86 | 0.85 | 0.79 |
| MINSQ | none | 0 | 0.86 | 0.88 | 0.86 | 0.88 | 0.86 | 0.88 | 0.86 | 0.88 |
|  | none | 4 | 0.89 | 0.90 | 0.89 | 0.90 | 0.89 | 0.90 | 0.89 | 0.90 |
|  | none | 8 | 0.89 | 0.89 | 0.89 | 0.89 | 0.89 | 0.89 | 0.89 | 0.89 |
|  | none | 16 | 0.88 | 0.89 | 0.88 | 0.89 | 0.88 | 0.89 | 0.88 | 0.89 |
|  | none | 32 | 0.86 | 0.87 | 0.86 | 0.87 | 0.86 | 0.87 | 0.86 | 0.87 |
|  | Hamming | 0 | 0.89 | 0.87 | 0.90 | 0.85 | 0.84 | 0.78 | 0.85 | 0.84 |
|  | Hamming | 4 | 0.86 | 0.81 | 0.85 | 0.82 | 0.86 | 0.83 | 0.90 | 0.86 |
|  | Hamming | 8 | 0.92 | 0.92 | 0.89 | 0.88 | 0.84 | 0.81 | 0.91 | 0.88 |
|  | Hamming | 16 | 0.89 | 0.85 | 0.90 | 0.80 | 0.86 | 0.75 | 0.82 | 0.81 |
|  | Hamming | 32 | 0.88 | 0.85 | 0.91 | 0.88 | 0.87 | 0.81 | 0.84 | 0.81 |
|  | Tukey | 0 | 0.91 | 0.91 | 0.89 | 0.86 | 0.86 | 0.78 | 0.86 | 0.86 |
|  | Tukey | 4 | 0.90 | 0.85 | 0.86 | 0.83 | 0.89 | 0.88 | 0.88 | 0.86 |
|  | Tukey | 8 | 0.88 | 0.90 | 0.89 | 0.88 | 0.85 | 0.85 | 0.88 | 0.81 |
|  | Tukey | 16 | 0.88 | 0.90 | 0.89 | 0.85 | 0.87 | 0.80 | 0.88 | 0.85 |
|  | Tukey | 32 | 0.90 | 0.90 | 0.88 | 0.86 | 0.83 | 0.73 | 0.87 | 0.87 |
|  | normalized | 0 | 0.86 | 0.83 | 0.84 | 0.81 | 0.86 | 0.83 | 0.84 | 0.86 |
|  | normalized | 4 | 0.90 | 0.90 | 0.92 | 0.89 | 0.90 | 0.90 | 0.92 | 0.90 |
|  | normalized | 8 | 0.90 | 0.87 | 0.91 | 0.91 | 0.90 | 0.90 | 0.89 | 0.87 |
|  | normalized | 16 | 0.89 | 0.86 | 0.89 | 0.87 | 0.90 | 0.86 | 0.86 | 0.81 |
|  | normalized | 32 | 0.89 | 0.86 | 0.87 | 0.83 | 0.89 | 0.86 | 0.86 | 0.85 |
| peak | none | 0 |  | 0.76 |  | 0.79 |  | 0.74 |  | 0.77 |
|  | none | 4 |  | 0.86 |  | 0.84 |  | 0.87 |  | 0.81 |
|  | none | 8 |  | 0.82 |  | 0.84 |  | 0.79 |  | 0.78 |
|  | none | 16 |  | 0.78 |  | 0.80 |  | 0.78 |  | 0.74 |
|  | none | 32 |  | 0.76 |  | 0.78 |  | 0.71 |  | 0.75 |
| area | none | 0 |  | 0.90 |  | 0.90 |  | 0.86 |  | 0.89 |
|  | none | 4 |  | 0.91 |  | 0.90 |  | 0.83 |  | 0.90 |
|  | none | 8 |  | 0.90 |  | 0.90 |  | 0.88 |  | 0.90 |
|  | none | 16 |  | 0.89 |  | 0.89 |  | 0.88 |  | 0.87 |
|  | none | 32 |  | 0.89 |  | 0.89 |  | 0.86 |  | 0.88 |
| Liesefeld A | none | 0 |  | 0.86 |  | 0.88 |  | 0.84 |  | 0.89 |
|  | none | 4 |  | 0.90 |  | 0.90 |  | 0.87 |  | 0.90 |
|  | none | 8 |  | 0.89 |  | 0.88 |  | 0.86 |  | 0.89 |
|  | none | 16 |  | 0.88 |  | 0.89 |  | 0.85 |  | 0.87 |
|  | none | 32 |  | 0.88 |  | 0.89 |  | 0.84 |  | 0.87 |
| Liesefeld B | none | 0 |  | 0.82 |  | 0.83 |  | 0.80 |  | 0.79 |
|  | none | 4 |  | 0.85 |  | 0.86 |  | 0.86 |  | 0.85 |
|  | none | 8 |  | 0.83 |  | 0.84 |  | 0.83 |  | 0.84 |
|  | none | 16 |  | 0.83 |  | 0.84 |  | 0.83 |  | 0.85 |
|  | none | 32 |  | 0.81 |  | 0.82 |  | 0.76 |  | 0.80 |
| *Note.* Reliability has been estimated by two-part coefficient alpha. The rows indicate combinations of similarity measures and weighting functions. The columns denote the measurement window and indicate if a penalty was used. MAXCOR and MINSQ refer to the template matching algorithms maximizing the correlation or minimizing the squared distance, respectively. peak refers to the peak latency approach, area to a standard 50% fractional area latency approach. Liesefeld A and Liesefeld B refer to modified fractional area latency approaches proposed by Liesefeld (2016; 2018). Liesefeld A uses 50% of the peak amplitude as the new baseline. Liesefeld B uses 30% of the peak amplitude as the baseline and additionally constrains the measurement window by the on- and offset of the component. | | | | | | | | | | |

## Validity

**Table A5.**

*Validity of different algorithms by task*

| approach | weight | task | [200 700] | | [250 700] | | [250 900] | | [300 600] | |
| --- | --- | --- | --- | --- | --- | --- | --- | --- | --- | --- |
|  |  |  | penalized | none | penalized | none | penalized | none | penalized | none |
| MAXCOR | none | flanker | 0.89 | 0.89 | 0.89 | 0.89 | 0.89 | 0.89 | 0.89 | 0.89 |
|  | none | nback | 0.80 | 0.79 | 0.80 | 0.79 | 0.80 | 0.79 | 0.80 | 0.79 |
|  | none | switching | 0.71 | 0.71 | 0.71 | 0.71 | 0.71 | 0.71 | 0.72 | 0.72 |
|  | Hamming | flanker | 0.92 | 0.91 | 0.91 | 0.91 | 0.88 | 0.88 | 0.87 | 0.84 |
|  | Hamming | nback | 0.87 | 0.87 | 0.76 | 0.77 | 0.77 | 0.73 | 0.58 | 0.60 |
|  | Hamming | switching | 0.81 | 0.76 | 0.82 | 0.76 | 0.75 | 0.75 | 0.70 | 0.67 |
|  | Tukey | flanker | 0.92 | 0.91 | 0.92 | 0.92 | 0.91 | 0.91 | 0.88 | 0.85 |
|  | Tukey | nback | 0.86 | 0.86 | 0.76 | 0.78 | 0.74 | 0.73 | 0.59 | 0.58 |
|  | Tukey | switching | 0.78 | 0.74 | 0.80 | 0.67 | 0.78 | 0.73 | 0.67 | 0.65 |
|  | normalized | flanker | 0.93 | 0.93 | 0.93 | 0.93 | 0.93 | 0.93 | 0.92 | 0.92 |
|  | normalized | nback | 0.86 | 0.88 | 0.90 | 0.89 | 0.89 | 0.88 | 0.88 | 0.86 |
|  | normalized | switching | 0.88 | 0.87 | 0.88 | 0.87 | 0.87 | 0.82 | 0.87 | 0.89 |
| MINSQ | none | flanker | 0.83 | 0.86 | 0.83 | 0.86 | 0.83 | 0.86 | 0.82 | 0.86 |
|  | none | nback | 0.75 | 0.77 | 0.75 | 0.77 | 0.75 | 0.77 | 0.75 | 0.77 |
|  | none | switching | 0.64 | 0.72 | 0.64 | 0.72 | 0.64 | 0.72 | 0.64 | 0.72 |
|  | Hamming | flanker | 0.90 | 0.90 | 0.88 | 0.87 | 0.72 | 0.69 | 0.87 | 0.87 |
|  | Hamming | nback | 0.83 | 0.83 | 0.81 | 0.80 | 0.71 | 0.71 | 0.79 | 0.69 |
|  | Hamming | switching | 0.84 | 0.81 | 0.87 | 0.79 | 0.68 | 0.67 | 0.76 | 0.71 |
|  | Tukey | flanker | 0.89 | 0.89 | 0.89 | 0.89 | 0.85 | 0.83 | 0.87 | 0.84 |
|  | Tukey | nback | 0.78 | 0.82 | 0.81 | 0.82 | 0.74 | 0.68 | 0.78 | 0.76 |
|  | Tukey | switching | 0.83 | 0.75 | 0.80 | 0.77 | 0.65 | 0.66 | 0.72 | 0.71 |
|  | normalized | flanker | 0.93 | 0.93 | 0.93 | 0.92 | 0.93 | 0.93 | 0.93 | 0.92 |
|  | normalized | nback | 0.88 | 0.88 | 0.89 | 0.88 | 0.87 | 0.87 | 0.88 | 0.83 |
|  | normalized | switching | 0.87 | 0.86 | 0.87 | 0.86 | 0.87 | 0.84 | 0.85 | 0.82 |
| peak | none | flanker |  | 0.86 |  | 0.88 |  | 0.86 |  | 0.85 |
|  | none | nback |  | 0.83 |  | 0.84 |  | 0.79 |  | 0.73 |
|  | none | switching |  | 0.81 |  | 0.84 |  | 0.84 |  | 0.77 |
| area | none | flanker |  | 0.84 |  | 0.81 |  | 0.66 |  | 0.67 |
|  | none | nback |  | 0.83 |  | 0.80 |  | 0.67 |  | 0.69 |
|  | none | switching |  | 0.72 |  | 0.74 |  | 0.76 |  | 0.59 |
| Liesefeld A | none | flanker |  | 0.93 |  | 0.92 |  | 0.88 |  | 0.86 |
|  | none | nback |  | 0.91 |  | 0.91 |  | 0.79 |  | 0.85 |
|  | none | switching |  | 0.82 |  | 0.87 |  | 0.86 |  | 0.77 |
| Liesefeld B | none | flanker |  | 0.80 |  | 0.82 |  | 0.82 |  | 0.85 |
|  | none | nback |  | 0.77 |  | 0.78 |  | 0.74 |  | 0.75 |
|  | none | switching |  | 0.78 |  | 0.83 |  | 0.82 |  | 0.79 |
| *Note.* Intra-class correlations focusing on absolute agreement. The rows indicate combinations of similarity measures and weighting functions. The columns denote the measurement window and indicate if a penalty was used. MAXCOR and MINSQ refer to the template matching algorithms maximizing the correlation or minimizing the squared distance, respectively. peak refers to the peak latency approach, area to a standard 50% fractional area latency approach. Liesefeld A and Liesefeld B refer to modified fractional area latency approaches proposed by Liesefeld (2016; 2018). Liesefeld A uses 50% of the peak amplitude as the new baseline. Liesefeld B uses 30% of the peak amplitude as the baseline and additionally constrains the measurement window by the on- and offset of the component. | | | | | | | | | | |

**Table A6.**

*Validity of different algorithms by filter setting*

| approach | weight | filter | [200 700] | | [250 700] | | [250 900] | | [300 600] | |
| --- | --- | --- | --- | --- | --- | --- | --- | --- | --- | --- |
|  |  |  | penalized | none | penalized | none | penalized | none | penalized | none |
| MAXCOR | none | 0 | 0.79 | 0.78 | 0.79 | 0.78 | 0.79 | 0.78 | 0.79 | 0.78 |
|  | none | 4 | 0.80 | 0.79 | 0.80 | 0.79 | 0.80 | 0.79 | 0.80 | 0.79 |
|  | none | 8 | 0.80 | 0.80 | 0.80 | 0.80 | 0.80 | 0.80 | 0.80 | 0.80 |
|  | none | 16 | 0.81 | 0.80 | 0.81 | 0.80 | 0.81 | 0.80 | 0.81 | 0.81 |
|  | none | 32 | 0.81 | 0.81 | 0.81 | 0.81 | 0.81 | 0.81 | 0.82 | 0.81 |
|  | Hamming | 0 | 0.84 | 0.82 | 0.81 | 0.80 | 0.80 | 0.79 | 0.68 | 0.67 |
|  | Hamming | 4 | 0.89 | 0.87 | 0.86 | 0.84 | 0.86 | 0.86 | 0.78 | 0.81 |
|  | Hamming | 8 | 0.87 | 0.87 | 0.82 | 0.80 | 0.80 | 0.80 | 0.74 | 0.67 |
|  | Hamming | 16 | 0.87 | 0.84 | 0.83 | 0.80 | 0.74 | 0.73 | 0.72 | 0.69 |
|  | Hamming | 32 | 0.86 | 0.85 | 0.82 | 0.82 | 0.78 | 0.76 | 0.66 | 0.69 |
|  | Tukey | 0 | 0.83 | 0.80 | 0.81 | 0.75 | 0.80 | 0.80 | 0.65 | 0.66 |
|  | Tukey | 4 | 0.88 | 0.87 | 0.87 | 0.84 | 0.87 | 0.86 | 0.82 | 0.81 |
|  | Tukey | 8 | 0.85 | 0.87 | 0.80 | 0.79 | 0.82 | 0.78 | 0.74 | 0.71 |
|  | Tukey | 16 | 0.85 | 0.84 | 0.81 | 0.80 | 0.76 | 0.73 | 0.67 | 0.67 |
|  | Tukey | 32 | 0.86 | 0.83 | 0.83 | 0.77 | 0.79 | 0.78 | 0.67 | 0.63 |
|  | normalized | 0 | 0.87 | 0.87 | 0.89 | 0.87 | 0.87 | 0.86 | 0.87 | 0.86 |
|  | normalized | 4 | 0.95 | 0.95 | 0.95 | 0.94 | 0.94 | 0.93 | 0.94 | 0.91 |
|  | normalized | 8 | 0.90 | 0.90 | 0.92 | 0.90 | 0.90 | 0.88 | 0.91 | 0.89 |
|  | normalized | 16 | 0.86 | 0.88 | 0.89 | 0.90 | 0.88 | 0.85 | 0.87 | 0.90 |
|  | normalized | 32 | 0.88 | 0.88 | 0.88 | 0.88 | 0.87 | 0.86 | 0.87 | 0.88 |
| MINSQ | none | 0 | 0.73 | 0.80 | 0.73 | 0.80 | 0.73 | 0.80 | 0.73 | 0.80 |
|  | none | 4 | 0.73 | 0.72 | 0.73 | 0.72 | 0.73 | 0.72 | 0.73 | 0.73 |
|  | none | 8 | 0.73 | 0.79 | 0.73 | 0.79 | 0.73 | 0.79 | 0.73 | 0.79 |
|  | none | 16 | 0.74 | 0.79 | 0.74 | 0.79 | 0.74 | 0.79 | 0.74 | 0.80 |
|  | none | 32 | 0.75 | 0.80 | 0.75 | 0.80 | 0.75 | 0.80 | 0.75 | 0.81 |
|  | Hamming | 0 | 0.85 | 0.82 | 0.84 | 0.83 | 0.72 | 0.73 | 0.82 | 0.76 |
|  | Hamming | 4 | 0.86 | 0.86 | 0.86 | 0.82 | 0.68 | 0.62 | 0.83 | 0.80 |
|  | Hamming | 8 | 0.86 | 0.87 | 0.86 | 0.83 | 0.70 | 0.72 | 0.78 | 0.71 |
|  | Hamming | 16 | 0.85 | 0.83 | 0.85 | 0.81 | 0.70 | 0.71 | 0.80 | 0.74 |
|  | Hamming | 32 | 0.86 | 0.84 | 0.85 | 0.81 | 0.71 | 0.68 | 0.80 | 0.77 |
|  | Tukey | 0 | 0.82 | 0.81 | 0.82 | 0.80 | 0.76 | 0.76 | 0.78 | 0.75 |
|  | Tukey | 4 | 0.85 | 0.83 | 0.85 | 0.87 | 0.74 | 0.70 | 0.81 | 0.81 |
|  | Tukey | 8 | 0.82 | 0.85 | 0.84 | 0.85 | 0.72 | 0.71 | 0.80 | 0.74 |
|  | Tukey | 16 | 0.83 | 0.83 | 0.82 | 0.83 | 0.74 | 0.71 | 0.76 | 0.75 |
|  | Tukey | 32 | 0.85 | 0.78 | 0.84 | 0.80 | 0.76 | 0.74 | 0.78 | 0.79 |
|  | normalized | 0 | 0.86 | 0.85 | 0.86 | 0.85 | 0.85 | 0.85 | 0.86 | 0.86 |
|  | normalized | 4 | 0.94 | 0.93 | 0.94 | 0.93 | 0.94 | 0.92 | 0.93 | 0.90 |
|  | normalized | 8 | 0.91 | 0.89 | 0.91 | 0.89 | 0.90 | 0.88 | 0.91 | 0.83 |
|  | normalized | 16 | 0.87 | 0.90 | 0.88 | 0.89 | 0.88 | 0.88 | 0.86 | 0.86 |
|  | normalized | 32 | 0.89 | 0.87 | 0.89 | 0.88 | 0.88 | 0.86 | 0.87 | 0.84 |
| peak | none | 0 |  | 0.78 |  | 0.83 |  | 0.77 |  | 0.80 |
|  | none | 4 |  | 0.90 |  | 0.90 |  | 0.91 |  | 0.79 |
|  | none | 8 |  | 0.84 |  | 0.85 |  | 0.82 |  | 0.77 |
|  | none | 16 |  | 0.83 |  | 0.86 |  | 0.84 |  | 0.77 |
|  | none | 32 |  | 0.80 |  | 0.83 |  | 0.82 |  | 0.78 |
| area | none | 0 |  | 0.78 |  | 0.78 |  | 0.70 |  | 0.66 |
|  | none | 4 |  | 0.83 |  | 0.80 |  | 0.71 |  | 0.64 |
|  | none | 8 |  | 0.80 |  | 0.78 |  | 0.69 |  | 0.64 |
|  | none | 16 |  | 0.78 |  | 0.77 |  | 0.67 |  | 0.65 |
|  | none | 32 |  | 0.80 |  | 0.79 |  | 0.71 |  | 0.66 |
| Liesefeld A | none | 0 |  | 0.87 |  | 0.90 |  | 0.81 |  | 0.82 |
|  | none | 4 |  | 0.90 |  | 0.91 |  | 0.91 |  | 0.85 |
|  | none | 8 |  | 0.90 |  | 0.90 |  | 0.84 |  | 0.84 |
|  | none | 16 |  | 0.87 |  | 0.89 |  | 0.82 |  | 0.83 |
|  | none | 32 |  | 0.87 |  | 0.89 |  | 0.83 |  | 0.81 |
| Liesefeld B | none | 0 |  | 0.72 |  | 0.80 |  | 0.75 |  | 0.81 |
|  | none | 4 |  | 0.79 |  | 0.79 |  | 0.81 |  | 0.76 |
|  | none | 8 |  | 0.78 |  | 0.79 |  | 0.78 |  | 0.79 |
|  | none | 16 |  | 0.81 |  | 0.83 |  | 0.81 |  | 0.78 |
|  | none | 32 |  | 0.81 |  | 0.83 |  | 0.81 |  | 0.83 |
| *Note.* Intra-class correlations focusing on absolute agreement. The rows indicate combinations of similarity measures and weighting functions. The columns denote the measurement window and indicate if a penalty was used. | | | | | | | | | | |

## Distribution of Fit Statistics

**Figure A1.**
*Distribution of Fit Statistics in the Empirical Study*


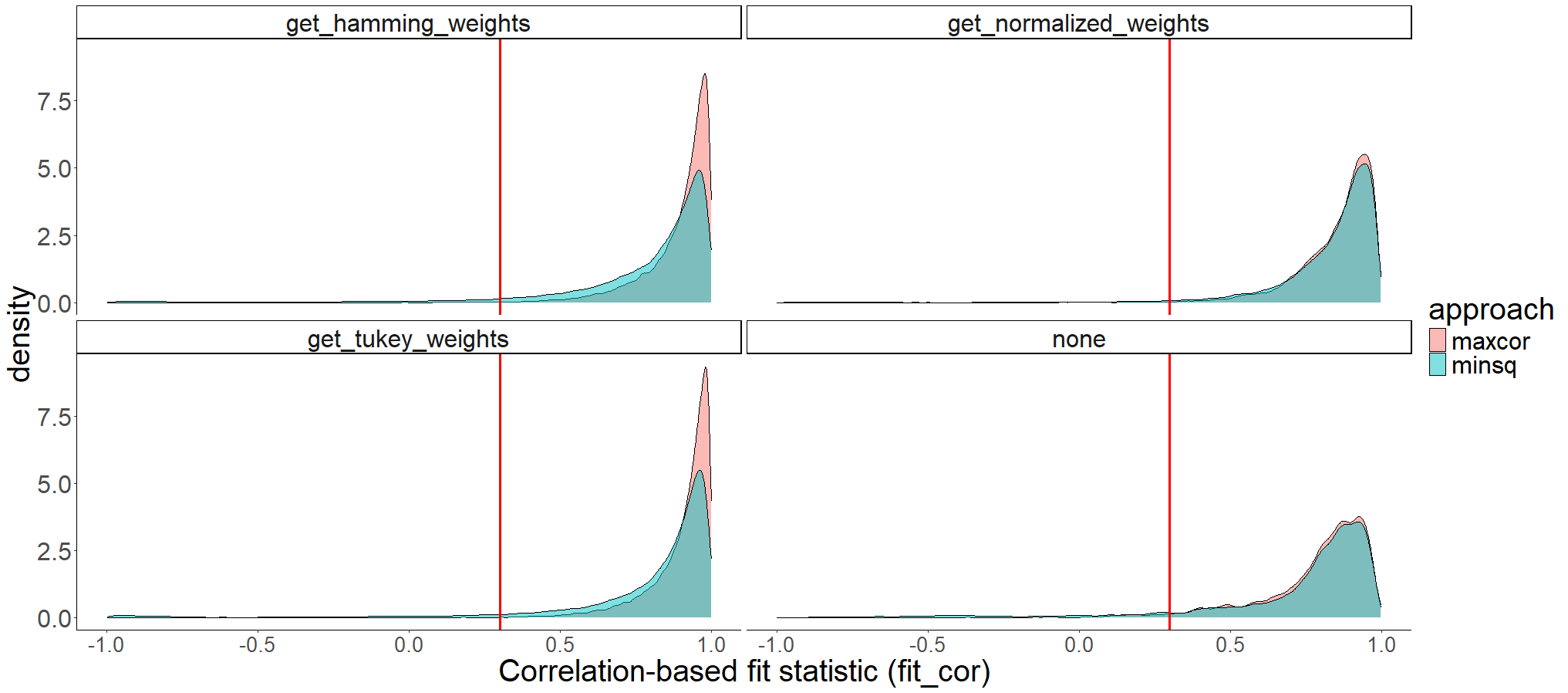

*Note.* Distribution of the fit statistic (fit_cor) in the simulation study across preprocessing steps and subjects by weighting function. MAXCOR and MINSQ refer to the template matching algorithms maximizing the correlation or minimizing the squared distance, respectively; the red line indicates the chosen cutoff of 0.3.

# Simulation

**Table A7.**
*Validity of different algorithms by filter setting*

| approach | weight | filter | [200 700] | | [250 700] | | [250 900] | | [300 600] | |
| --- | --- | --- | --- | --- | --- | --- | --- | --- | --- | --- |
|  |  |  | penalized | none | penalized | none | penalized | none | penalized | none |
| MAXCOR | none | 4 | 0.93 | 0.94 | 0.93 | 0.95 | 0.92 | 0.95 | 0.91 | 0.95 |
|  | none | 8 | 0.95 | 0.88 | 0.95 | 0.95 | 0.95 | 0.93 | 0.96 | 0.93 |
|  | none | 16 | 0.94 | 0.95 | 0.95 | 0.94 | 0.92 | 0.95 | 0.94 | 0.95 |
|  | none | 32 | 0.95 | 0.93 | 0.93 | 0.95 | 0.93 | 0.97 | 0.92 | 0.97 |
|  | Hamming | 4 | 0.83 | 0.58 | 0.79 | 0.63 | 0.78 | 0.75 | 0.48 | 0.35 |
|  | Hamming | 8 | 0.48 | 0.20 | 0.53 | 0.26 | 0.77 | 0.70 | 0.11 | 0.09 |
|  | Hamming | 16 | 0.26 | 0.23 | 0.41 | 0.50 | 0.79 | 0.80 | 0.12 | 0.05 |
|  | Hamming | 32 | 0.57 | 0.34 | 0.57 | 0.45 | 0.79 | 0.82 | 0.15 | 0.05 |
|  | Tukey | 4 | 0.86 | 0.70 | 0.79 | 0.52 | 0.78 | 0.76 | 0.49 | 0.35 |
|  | Tukey | 8 | 0.63 | 0.25 | 0.56 | 0.31 | 0.73 | 0.74 | 0.20 | 0.12 |
|  | Tukey | 16 | 0.53 | 0.19 | 0.57 | 0.23 | 0.86 | 0.84 | 0.13 | 0.09 |
|  | Tukey | 32 | 0.55 | 0.20 | 0.51 | 0.26 | 0.85 | 0.88 | 0.17 | 0.03 |
|  | normalized | 4 | 0.97 | 0.97 | 0.96 | 0.96 | 0.95 | 0.96 | 0.90 | 0.85 |
|  | normalized | 8 | 0.93 | 0.62 | 0.93 | 0.82 | 0.83 | 0.55 | 0.95 | 0.88 |
|  | normalized | 16 | 0.50 | 0.83 | 0.64 | 0.82 | 0.57 | 0.89 | 0.79 | 0.72 |
|  | normalized | 32 | 0.88 | 0.82 | 0.91 | 0.82 | 0.92 | 0.81 | 0.81 | 0.66 |
| MINSQ | none | 4 | 0.80 | 0.81 | 0.81 | 0.85 | 0.78 | 0.80 | 0.80 | 0.85 |
|  | none | 8 | 0.89 | 0.86 | 0.85 | 0.88 | 0.82 | 0.88 | 0.82 | 0.88 |
|  | none | 16 | 0.85 | 0.91 | 0.87 | 0.92 | 0.86 | 0.90 | 0.85 | 0.90 |
|  | none | 32 | 0.92 | 0.94 | 0.91 | 0.92 | 0.89 | 0.91 | 0.90 | 0.92 |
|  | Hamming | 4 | 0.73 | 0.71 | 0.68 | 0.63 | 0.72 | 0.79 | 0.54 | 0.48 |
|  | Hamming | 8 | 0.72 | 0.39 | 0.67 | 0.58 | 0.73 | 0.62 | 0.55 | 0.48 |
|  | Hamming | 16 | 0.76 | 0.65 | 0.71 | 0.68 | 0.74 | 0.84 | 0.54 | 0.54 |
|  | Hamming | 32 | 0.75 | 0.70 | 0.62 | 0.59 | 0.82 | 0.86 | 0.58 | 0.31 |
|  | Tukey | 4 | 0.81 | 0.74 | 0.74 | 0.73 | 0.74 | 0.78 | 0.56 | 0.48 |
|  | Tukey | 8 | 0.75 | 0.50 | 0.70 | 0.64 | 0.76 | 0.68 | 0.52 | 0.43 |
|  | Tukey | 16 | 0.78 | 0.71 | 0.72 | 0.75 | 0.78 | 0.84 | 0.59 | 0.49 |
|  | Tukey | 32 | 0.83 | 0.76 | 0.59 | 0.57 | 0.82 | 0.88 | 0.56 | 0.48 |
|  | normalized | 4 | 0.95 | 0.97 | 0.96 | 0.98 | 0.98 | 0.96 | 0.91 | 0.90 |
|  | normalized | 8 | 0.96 | 0.91 | 0.96 | 0.92 | 0.97 | 0.65 | 0.72 | 0.92 |
|  | normalized | 16 | 0.83 | 0.97 | 0.85 | 0.97 | 0.72 | 0.97 | 0.82 | 0.92 |
|  | normalized | 32 | 0.95 | 0.94 | 0.95 | 0.92 | 0.97 | 0.96 | 0.89 | 0.92 |
| peak | none | 4 |  | 0.94 |  | 0.91 |  | 0.93 |  | 0.72 |
|  | none | 8 |  | 0.52 |  | 0.43 |  | 0.75 |  | 0.20 |
|  | none | 16 |  | 0.49 |  | 0.57 |  | 0.86 |  | 0.25 |
|  | none | 32 |  | 0.61 |  | 0.55 |  | 0.67 |  | 0.35 |
| area | none | 4 |  | 0.64 |  | 0.58 |  | 0.74 |  | 0.46 |
|  | none | 8 |  | 0.71 |  | 0.65 |  | 0.66 |  | 0.40 |
|  | none | 16 |  | 0.69 |  | 0.66 |  | 0.55 |  | 0.38 |
|  | none | 32 |  | 0.67 |  | 0.62 |  | 0.66 |  | 0.51 |
| Liesefeld A | none | 4 |  | 0.95 |  | 0.94 |  | 0.92 |  | 0.69 |
|  | none | 8 |  | 0.89 |  | 0.88 |  | 0.86 |  | 0.54 |
|  | none | 16 |  | 0.82 |  | 0.82 |  | 0.67 |  | 0.37 |
|  | none | 32 |  | 0.84 |  | 0.76 |  | 0.88 |  | 0.40 |
| Liesefeld B | none | 4 |  | 0.91 |  | 0.89 |  | 0.83 |  | 0.88 |
|  | none | 8 |  | 0.41 |  | 0.44 |  | 0.75 |  | 0.40 |
|  | none | 16 |  | 0.60 |  | 0.54 |  | 0.65 |  | 0.21 |
|  | none | 32 |  | 0.70 |  | 0.78 |  | 0.65 |  | 0.85 |
| *Note.* Intra-class correlations focusing on absolute agreement. The rows indicate combinations of similarity measures and weighting functions. The columns denote the measurement window and indicate if a penalty was used. MAXCOR and MINSQ refer to the template matching algorithms maximizing the correlation or minimizing the squared distance, respectively. peak refers to the peak latency approach, area to a standard 50% fractional area latency approach. Liesefeld A and Liesefeld B refer to modified fractional area latency approaches proposed by Liesefeld (2016; 2018). Liesefeld A uses 50% of the peak amplitude as the new baseline. Liesefeld B uses 30% of the peak amplitude as the baseline and additionally constrains the measurement window by the on- and offset of the component. | | | | | | | | | | |

## Distribution of Fit Statistics

**Figure A2.**
*Distribution of Fit Statistics in the Simulation*


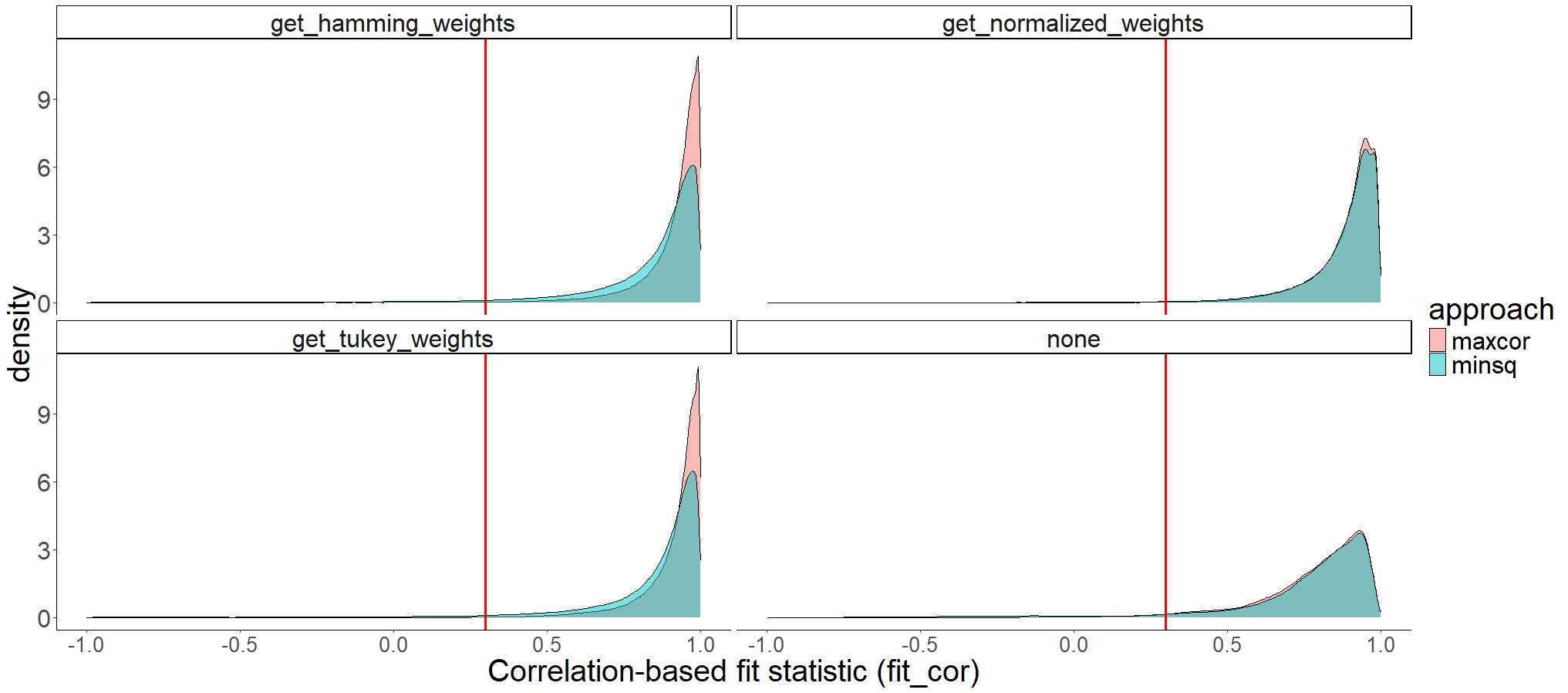

*Note.* Distribution of the fit statistic (fit_cor) in the simulation study across preprocessing steps and subjects by weighting function. MAXCOR and MINSQ refer to the template matching algorithms maximizing the correlation or minimizing the squared distance, respectively; the red line indicates the chosen cutoff of 0.3.

# Simulation – Linear Shift

To complement the original simulation based on component stretching, we implemented an alternative simulation that models a linear shift in latency across participants, similar to the approach described by Kiesel et al. (2008). This simulation aims to reflect a plausible pattern of latency variability in experimental data.

For each simulated participant, we drew a random latency shift from a normal distribution with a mean of 45 ms and a standard deviation of 15 ms. This shift was then applied to the ERP component in all of that participant’s experimental trials. To implement the temporal shift with sub-sample precision, we used spline interpolation, allowing us to delay the signal by arbitrary amounts not constrained to integer multiples of the sampling interval.

Following the shift, values between 0 and the shift duration in the transformed signal were set to 0. All other aspects of the simulation remained identical to the original stretching-based simulation pipeline.

**Table A8.** Mean v*alidity of different algorithms (linear shift)*

| approach | weight | [200 700] | | [250 700] | | [250 900] | | [300 600] | |
| --- | --- | --- | --- | --- | --- | --- | --- | --- | --- |
|  |  | penalized | none | penalized | none | penalized | none | penalized | none |
| MAXCOR | none | 0.89 [0.84, 0.94] | 0.88 [0.79, 0.95] | 0.89 [0.83, 0.95] | 0.88 [0.82, 0.94] | 0.89 [0.82, 0.94] | 0.91 [0.86, 0.96] | 0.90 [0.84, 0.94] | 0.88 [0.82, 0.94] |
|  | Hamming | 0.61 [0.45, 0.80] | 0.54 [0.38, 0.79] | 0.62 [0.47, 0.79] | 0.58 [0.44, 0.79] | 0.69 [0.58, 0.79] | 0.65 [0.51, 0.79] | 0.30 [0.22, 0.43] | 0.26 [0.18, 0.38] |
|  | Tukey | 0.67 [0.54, 0.80] | 0.59 [0.48, 0.74] | 0.54 [0.39, 0.73] | 0.54 [0.39, 0.75] | 0.63 [0.44, 0.81] | 0.61 [0.42, 0.80] | 0.28 [0.20, 0.39] | 0.23 [0.15, 0.36] |
|  | normalized | 0.93 [0.88, 0.96] | 0.88 [0.79, 0.96] | 0.89 [0.81, 0.95] | 0.90 [0.85, 0.94] | 0.89 [0.82, 0.94] | 0.77 [0.65, 0.92] | 0.90 [0.84, 0.94] | 0.83 [0.75, 0.91] |
| MINSQ | none | 0.88 [0.82, 0.92] | 0.88 [0.79, 0.93] | 0.86 [0.80, 0.90] | 0.88 [0.79, 0.94] | 0.87 [0.82, 0.91] | 0.88 [0.79, 0.93] | 0.82 [0.75, 0.89] | 0.89 [0.81, 0.93] |
|  | Hamming | 0.61 [0.48, 0.73] | 0.54 [0.40, 0.69] | 0.52 [0.35, 0.68] | 0.45 [0.25, 0.65] | 0.62 [0.51, 0.72] | 0.65 [0.55, 0.73] | 0.36 [0.21, 0.52] | 0.34 [0.19, 0.51] |
|  | Tukey | 0.66 [0.52, 0.78] | 0.64 [0.49, 0.77] | 0.48 [0.28, 0.68] | 0.52 [0.33, 0.70] | 0.61 [0.46, 0.74] | 0.60 [0.41, 0.76] | 0.37 [0.21, 0.55] | 0.31 [0.16, 0.51] |
|  | normalized | 0.93 [0.88, 0.96] | 0.89 [0.81, 0.95] | 0.90 [0.81, 0.96] | 0.89 [0.81, 0.95] | 0.92 [0.85, 0.96] | 0.88 [0.81, 0.94] | 0.89 [0.82, 0.94] | 0.88 [0.78, 0.95] |
| peak | none |  | 0.73 [0.62, 0.90] |  | 0.71 [0.53, 0.88] |  | 0.71 [0.51, 0.91] |  | 0.51 [0.34, 0.72] |
| area | none |  | 0.56 [0.41, 0.71] |  | 0.57 [0.41, 0.72] |  | 0.59 [0.46, 0.7] |  | 0.36 [0.24, 0.49] |
| Liesefeld A | none |  | 0.86 [0.77, 0.92] |  | 0.83 [0.72, 0.92] |  | 0.80 [0.69, 0.89] |  | 0.59 [0.42, 0.74] |
| Liesefeld B | none |  | 0.72 [0.61, 0.84] |  | 0.67 [0.47, 0.86] |  | 0.69 [0.50, 0.87] |  | 0.76 [0.65, 0.85] |
| Note. Intra-class correlations focusing on absolute agreement. Values in brackets provide bootstrapped 95% confidence intervals. The rows indicate combinations of similarity measure and weighting function. The columns denote the measurement window and indicate if a penalty was used. MAXCOR and MINSQ refer to the template matching algorithms maximizing the correlation or minimizing the squared distance, respectively. peak refers to the peak latency approach, area to a standard 50% fractional area latency approach. Liesefeld A refers to a modified fractional area latency approach, using 50% of the peak amplitude as the new baseline and Liesefeld B uses 30% of the peak amplitude as the baseline and additionally constrains the measurement window by the on- and offset of the component. | | | | | | | | | |

**Table A9.** *Validity of different algorithms by filter setting (linear shift)*

| approach | weight | filter | [200 700] | | [250 700] | | [250 900] | | [300 600] | |
| --- | --- | --- | --- | --- | --- | --- | --- | --- | --- | --- |
|  |  |  | penalized | none | penalized | none | penalized | none | penalized | none |
| MAXCOR | none | 4 | 0.89 | 0.93 | 0.92 | 0.92 | 0.91 | 0.94 | 0.93 | 0.92 |
|  | none | 8 | 0.94 | 0.85 | 0.91 | 0.89 | 0.91 | 0.93 | 0.94 | 0.92 |
|  | none | 16 | 0.86 | 0.86 | 0.87 | 0.88 | 0.90 | 0.90 | 0.86 | 0.88 |
|  | none | 32 | 0.88 | 0.86 | 0.88 | 0.83 | 0.83 | 0.88 | 0.85 | 0.81 |
|  | Hamming | 4 | 0.84 | 0.82 | 0.82 | 0.82 | 0.74 | 0.71 | 0.69 | 0.65 |
|  | Hamming | 8 | 0.66 | 0.46 | 0.56 | 0.56 | 0.66 | 0.61 | 0.21 | 0.15 |
|  | Hamming | 16 | 0.47 | 0.46 | 0.63 | 0.53 | 0.68 | 0.66 | 0.12 | 0.15 |
|  | Hamming | 32 | 0.45 | 0.41 | 0.45 | 0.42 | 0.67 | 0.63 | 0.20 | 0.09 |
|  | Tukey | 4 | 0.85 | 0.86 | 0.79 | 0.74 | 0.68 | 0.64 | 0.67 | 0.57 |
|  | Tukey | 8 | 0.72 | 0.53 | 0.56 | 0.52 | 0.55 | 0.53 | 0.17 | 0.14 |
|  | Tukey | 16 | 0.57 | 0.57 | 0.44 | 0.44 | 0.62 | 0.59 | 0.12 | 0.09 |
|  | Tukey | 32 | 0.52 | 0.42 | 0.37 | 0.46 | 0.67 | 0.67 | 0.13 | 0.12 |
|  | normalized | 4 | 0.97 | 0.96 | 0.94 | 0.97 | 0.96 | 0.96 | 0.86 | 0.90 |
|  | normalized | 8 | 0.97 | 0.92 | 0.86 | 0.88 | 0.96 | 0.79 | 0.94 | 0.88 |
|  | normalized | 16 | 0.94 | 0.89 | 0.89 | 0.88 | 0.81 | 0.68 | 0.90 | 0.84 |
|  | normalized | 32 | 0.84 | 0.76 | 0.86 | 0.88 | 0.83 | 0.64 | 0.88 | 0.71 |
| MINSQ | none | 4 | 0.89 | 0.90 | 0.85 | 0.92 | 0.87 | 0.88 | 0.71 | 0.87 |
|  | none | 8 | 0.89 | 0.83 | 0.85 | 0.87 | 0.83 | 0.87 | 0.87 | 0.91 |
|  | none | 16 | 0.87 | 0.93 | 0.90 | 0.89 | 0.89 | 0.88 | 0.85 | 0.89 |
|  | none | 32 | 0.85 | 0.85 | 0.83 | 0.85 | 0.90 | 0.90 | 0.85 | 0.87 |
|  | Hamming | 4 | 0.62 | 0.63 | 0.57 | 0.50 | 0.67 | 0.68 | 0.34 | 0.36 |
|  | Hamming | 8 | 0.56 | 0.40 | 0.52 | 0.38 | 0.59 | 0.52 | 0.33 | 0.34 |
|  | Hamming | 16 | 0.64 | 0.56 | 0.55 | 0.52 | 0.57 | 0.66 | 0.35 | 0.31 |
|  | Hamming | 32 | 0.64 | 0.59 | 0.46 | 0.42 | 0.66 | 0.74 | 0.41 | 0.36 |
|  | Tukey | 4 | 0.65 | 0.68 | 0.58 | 0.52 | 0.63 | 0.63 | 0.38 | 0.32 |
|  | Tukey | 8 | 0.61 | 0.52 | 0.47 | 0.56 | 0.59 | 0.52 | 0.33 | 0.24 |
|  | Tukey | 16 | 0.70 | 0.66 | 0.53 | 0.56 | 0.60 | 0.56 | 0.37 | 0.33 |
|  | Tukey | 32 | 0.68 | 0.70 | 0.36 | 0.46 | 0.63 | 0.68 | 0.40 | 0.35 |
|  | normalized | 4 | 0.96 | 0.96 | 0.95 | 0.96 | 0.94 | 0.95 | 0.85 | 0.85 |
|  | normalized | 8 | 0.94 | 0.86 | 0.93 | 0.84 | 0.95 | 0.87 | 0.91 | 0.92 |
|  | normalized | 16 | 0.92 | 0.85 | 0.91 | 0.86 | 0.87 | 0.85 | 0.91 | 0.90 |
|  | normalized | 32 | 0.90 | 0.87 | 0.81 | 0.90 | 0.93 | 0.86 | 0.89 | 0.83 |
| peak | none | 4 |  | 0.95 |  | 0.89 |  | 0.94 |  | 0.57 |
|  | none | 8 |  | 0.42 |  | 0.59 |  | 0.71 |  | 0.45 |
|  | none | 16 |  | 0.87 |  | 0.73 |  | 0.54 |  | 0.49 |
|  | none | 32 |  | 0.67 |  | 0.62 |  | 0.64 |  | 0.53 |
| area | none | 4 |  | 0.54 |  | 0.55 |  | 0.65 |  | 0.35 |
|  | none | 8 |  | 0.63 |  | 0.59 |  | 0.56 |  | 0.38 |
|  | none | 16 |  | 0.57 |  | 0.56 |  | 0.52 |  | 0.35 |
|  | none | 32 |  | 0.51 |  | 0.58 |  | 0.63 |  | 0.38 |
| Liesefeld A | none | 4 |  | 0.83 |  | 0.82 |  | 0.84 |  | 0.58 |
|  | none | 8 |  | 0.91 |  | 0.87 |  | 0.74 |  | 0.65 |
|  | none | 16 |  | 0.87 |  | 0.78 |  | 0.81 |  | 0.50 |
|  | none | 32 |  | 0.82 |  | 0.85 |  | 0.81 |  | 0.62 |
| Liesefeld B | none | 4 |  | 0.84 |  | 0.81 |  | 0.84 |  | 0.85 |
|  | none | 8 |  | 0.55 |  | 0.66 |  | 0.77 |  | 0.71 |
|  | none | 16 |  | 0.69 |  | 0.56 |  | 0.54 |  | 0.74 |
|  | none | 32 |  | 0.82 |  | 0.65 |  | 0.60 |  | 0.75 |
| *Note.* Intra-class correlations focusing on absolute agreement. The rows indicate combinations of similarity measures and weighting functions. The columns denote the measurement window and indicate if a penalty was used. MAXCOR and MINSQ refer to the template matching algorithms maximizing the correlation or minimizing the squared distance, respectively. peak refers to the peak latency approach, area to a standard 50% fractional area latency approach. Liesefeld A and Liesefeld B refer to modified fractional area latency approaches proposed by Liesefeld (2016; 2018). Liesefeld A uses 50% of the peak amplitude as the new baseline. Liesefeld B uses 30% of the peak amplitude as the baseline and additionally constrains the measurement window by the on- and offset of the component. | | | | | | | | | | |

**Figure A3***ICC estimates by extraction method - Linear Shift
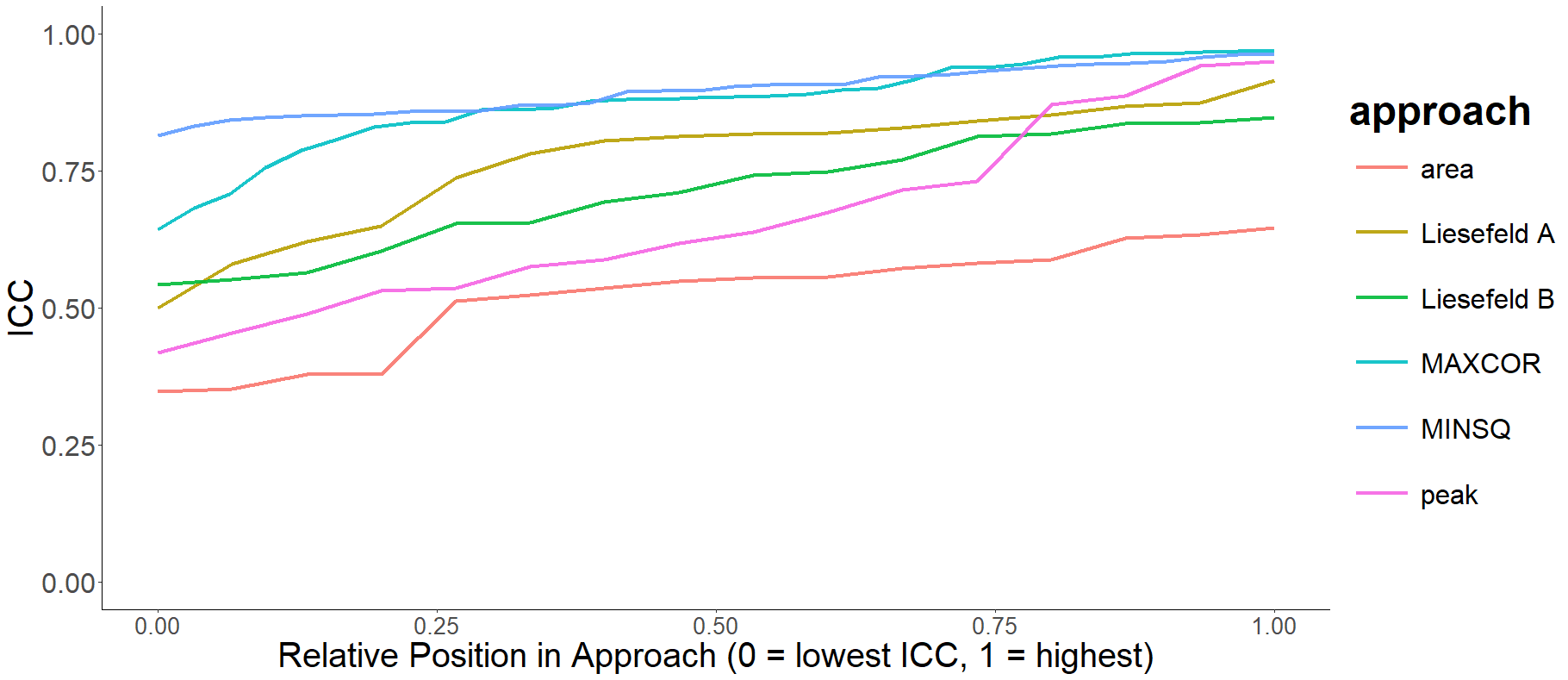
*

*Note.* Overview of ICC focusing on absolute agreement between simulated shift and recovered shift by latency extraction method. The plot shows all ICC estimates across tasks, preprocessing settings and weighting windows ordered by size within a given approach. For the template matching approaches, only the normalized weighting function is displayed. MAXCOR and MINSQ refer to the template matching algorithms maximizing the correlation or minimizing the squared distance, respectively. peak refers to the peak latency approach, area to a standard 50% fractional area latency approach. Liesefeld A refers to a modified fractional area latency approach, using 50% of the peak amplitude as the new baseline and Liesefeld B uses 30% of the peak amplitude as the baseline and additionally constrains the measurement window by the on- and offset of the component.

## Distribution of Fit Statistics

**Figure A4.**
*Distribution of Fit Statistics in the Simulation*


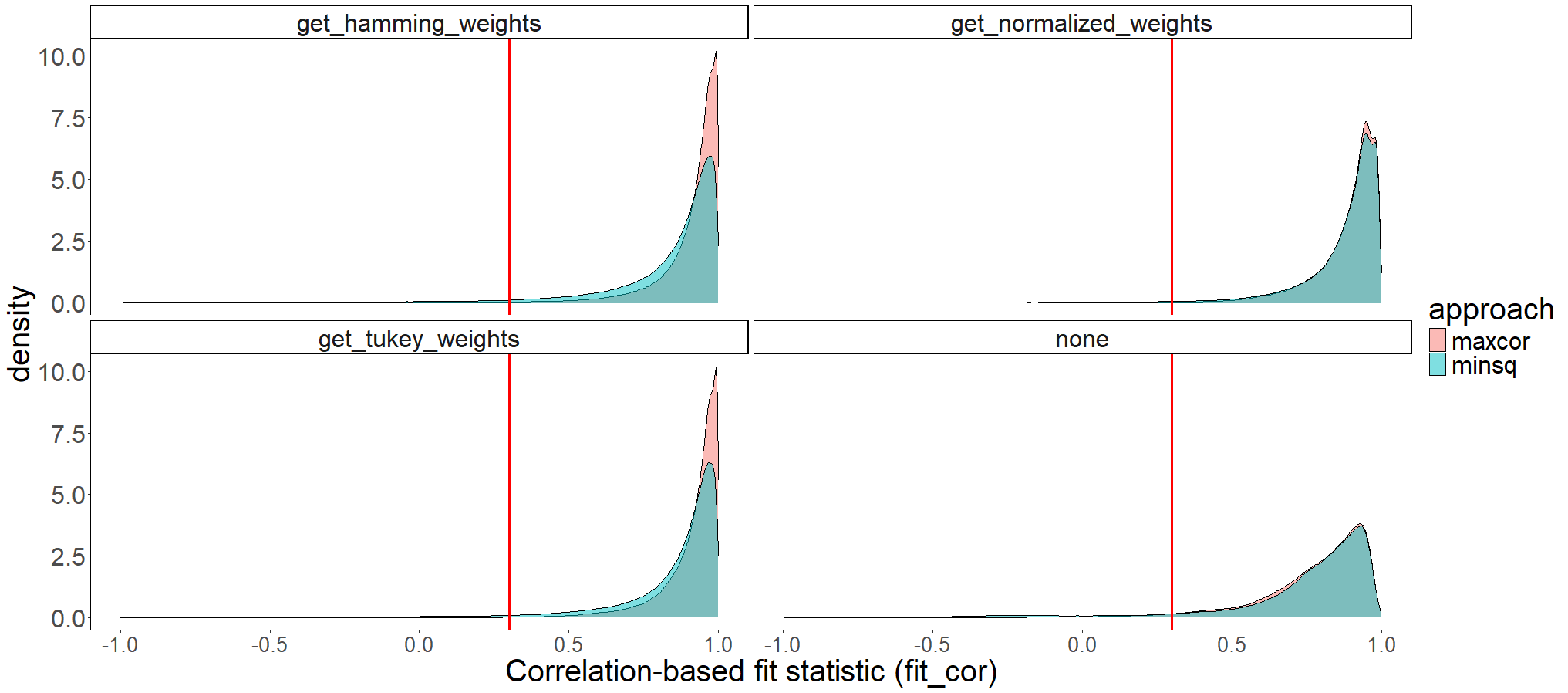

*Note.* Distribution of the fit statistic (fit_cor) in the simulation study across preprocessing steps and subjects by weighting function. MAXCOR and MINSQ refer to the template matching algorithms maximizing the correlation or minimizing the squared distance, respectively; the red line indicates the chosen cutoff of 0.3.
