# Supplementary material for: Exploring a Dynamic Template Matching Algorithm for the Automatic Extraction of P3 Latencies
Source: Psychophysiology. 2025 Dec 23;63(1):e70212. doi: 10.1111/psyp.70212 (PMC12728138; doi:10.1111/psyp.70212)
Supplement: Supplementary file 2 — Appendix S2: psyp70212‐sup‐0002‐Supplemental Materials.pdf. [file PSYP-63-e70212-s002.pdf]

Supplementary Material: Detailed Explanation of the Template Matching Algorithm

This supplement provides additional details on parameter settings, handling of the fit statistics, and edge cases. It also includes practical recommendations and limitations. The goal is to make design choices transparent and offer guidance for researchers interested in applying this method.

1. Parameter Settings

Many of the parameter choices—such as the minimum fit index, optimization bounds, and penalty functions—were based on reasonable assumptions and empirical experience rather than systematic optimization. Below we explain these settings and why they were chosen.

Optimization Bounds

- **Amplitude parameter (a):** [0.2, 20]
  These limits were set to avoid unrealistic amplitude transformations. Without bounds, the MINSQ algorithm sometimes converged to $a = 0$, which is clearly implausible (for example, when a positive component in the grand average appears only with negative amplitudes in subject-level ERPs).
- **Latency parameter (b):** [0.3, 2]
  These limits were set to avoid extreme latency shifts. Without bounds, the algorithm could overfit early or late components, especially when the data are noisy.
- **Impact:** The bounds prevent extreme values without interfering with normal estimation. Amplitude is unlikely to differ from the grand average by more than a factor of 5, and latency is unlikely to deviate by more than a factor of 2. If larger deviations are expected, these bounds can be adjusted.

Penalty Function

- **Purpose:** The penalty discourages convergence on unrealistic parameter values when the ERP morphology is ambiguous. Unlike the hard limits from the bounds, the penalty only reduces the likelihood of settling on implausible regions.
- **Implementation:** An exponential penalty is applied if $b \leq 0.66$ or $b \geq 1.5$.
- **Impact:** This reduces extreme solutions and improves the proportion of reasonable fits without compromising reliability or correlation with the reference measure. Since we do not want to impact the algorithm in plausible regions of parameter space, we applied no penalty for b values around 1.

- **Limitation:** The penalty is a pragmatic solution rather than an optimized one. More sophisticated approaches could perform better.

Weighting Windows

- **Purpose:** The window emphasizes the region of interest (e.g., the P3) while still including surrounding context.
- **Impact:** The size of the window had little impact as long as it fully captured the component.
- **Recommendation:** Use a window that covers most of the component activity observed in the grand average, similar to area-based measures (e.g., 250–700 ms for the P3 in most cases).

Weighting Functions

- **Purpose:** The exact specification of the weighting vector aims to place additional emphasis on specific parts of the component within the weighting window. Not all activity within the measurement window is equally important, but we try to incorporate information of activity from surrounding components into our judgment when extracting component latencies. A weighting function allows broader weighting windows that incorporate more information, but weigh this information accordingly.
- **Implementation:** We tested several functions. These were uninformed by the grand average structure, e.g. no weights, a Tukey weighting function, or a Hamming weighting function. Additionally, we tested a weighting function that places additional importance on the peak of the component by using the maximum-normalized grand average amplitude as a weighting function.
- **Recommendation:** Use the maximum-normalized grand average amplitude as a weighting function. This gives maximum emphasis to the peak and gradually taper toward the component edges. The other weighting functions did not perform as well.
- **Limitation**: As the present study only investigated the P3, we do not know how these findings regarding the weighting function will generalize to other components. We believe that the weighting function should reflect human behavior. In the case of the P3, this seems to be a weighting function that places additional importance on the peak of the component. For earlier components, the weighting function may have to be independent of the amplitude in the grand average, as this would likely place too much emphasis on later parts of the signal. We are eager to extend our approach to other components and test this assumption formally.

Similarity Measure

Based on exploratory results:

- MINSQ (minimize squared distance), combined with the penalty function and a normalized weighting vector, provided the most robust performance in our tests.

Important note: These recommendations come from exploratory analysis of only three tasks and should be considered provisional. More research is needed before these can be treated as default settings.

2. Fit Statistics Handling

The fit statistics measure morphological similarity between the transformed template and the ERP. They also serve as a quality control metric:

- **Automatic rejection:**
  $r < .30$ → discard automatically. In our data, latencies from such cases were almost never meaningful.
- **Manual review recommended:**
  $.30 \le r < .60$ → visual inspection suggested. These ERPs often contained noise or overlapping components but sometimes yielded valid estimates.

Why these thresholds? They were chosen pragmatically to balance efficiency and accuracy, based on extensive empirical review of ERP–fit pairs. They are not universal. Researchers should examine fit statistics distributions in their own data before choosing thresholds.

For future work, we recommend systematic evaluation of the relationship between fit statistic values and actual estimation accuracy to establish more principled cutoffs.

3. Edge Cases: Multiple Peaks and Wrong Matches

ERPs sometimes have multiple peaks or overlapping components in the region of interest.

- **Algorithm behavior:** The algorithm aligns the template to maximize overall similarity within the weighted window. The behavior in cases with multiple peaks is not easily generalized, as it depends on the ERP shape. Optimizing for similarity might lead to:
  - Selecting the earlier or later peak, depending on the similarity of each peak and the surrounding activity (especially if the peaks are relatively far apart from each other)
  - Selecting "both peaks", if both are reflected in the grand average
  - Or settling on an intermediate alignment between the peak (MINSQ will tend to do this more than MAXCOR, as this intermediate alignment tends to hurt correlations more than distance metrics)

- **Manual intervention:** Ambiguous cases often produce lower fit statistics and are flagged for review using the thresholds mentioned above.

4. Generalizability to Other Components and Paradigms

While our findings show that template matching works well for the P3 component in the tasks tested, generalizability is still an open question. Below are some factors to consider.

Variability within the P3

The P3 is not uniform. Its shape, amplitude, and duration can vary across paradigms, populations, and conditions. For example:

- Some P3s are broad and slow, others are sharp and brief.
- Some overlap minimally with other components, while others occur in crowded temporal regions.

These differences can affect how well the algorithm aligns the template.

Findings from Our Dataset

We included three tasks that produced diverse P3 shapes, from more sharply peaked to broadly distributed. This was intentional to test robustness. The algorithm performed well across these tasks, suggesting it can handle moderate variability in component shape and context.

The tasks we tested were relatively complex (Flanker, Nback, Switching), producing ERPs harder to parameterize than those in classic oddball paradigms. Good performance in these conditions gives us some confidence for simpler cases.

Other Components

Earlier components like N1 or P2 are harder to handle because they are narrower and surrounded by strong neighboring components. Overlap and low amplitude make them more challenging to match reliably. Template matching might still help in some cases, but this is untested and needs systematic evaluation.

5. Practical Guidance for Researchers

If you plan to apply template matching in a new context:

1. Start with the recommended settings (MINSQ + penalty + normalized weights) or small variations.
2. Visually inspect ERPs and their corresponding latency estimates across a range of fit statistics (from low to high). This helps you see whether the algorithm behaves

sensibly on your data.

3. Treat initial results as exploratory, especially when applying the method to components other than the P3 or to very different paradigms.

We are developing a dedicated tool to make visual inspection easier and to simplify the application of the template matching algorithm. You can find the current developmental version here: https://github.com/SLesche/template-matching-app
